# Curcumin as an Adjuvant to Cancer Immunotherapy

**DOI:** 10.3389/fonc.2021.675923

**Published:** 2021-08-16

**Authors:** Silpita Paul, Gaurisankar Sa

**Affiliations:** Division of Molecular Medicine, Bose Institute, Kolkata, India

**Keywords:** curcumin, dendritic cell, CTLA4, PD1/PD-L1, immune cells, Treg cells, immunotherapy

## Abstract

The components of the immune system play a very sincere and crucial role in combating tumors. However, despite their firm efforts of elimination, tumor cells cleverly escape the surveillance process by adopting several immune evasion mechanisms. The conversion of immunogenicity of tumor microenvironment into tolerogenic is considered as a prime reason for tumor immune escape. Therapeutically, different immunotherapies have been adopted to block such immune escaping routes along with better clinical outcomes. Still, the therapies are haunted by several drawbacks. Over time, curcumin has been considered as a potential anti-cancer molecule. Its potentialities have been recorded against the standard hallmarks of cancer such as continuous proliferation, escaping apoptosis, continuous angiogenesis, insensitivity to growth inhibitors, tissue invasion, and metastasis. Hence, the diversity of curcumin functioning has already been established and exploration of its application with immunotherapies might open up a new avenue for scientists and clinicians. In this review, we briefly discuss the tumor’s way of immune escaping, followed by various modern immunotherapies that have been used to encounter the escaping paths and their minute flaws. Finally, the conclusion has been drawn with the application of curcumin as a potential immune-adjuvant, which fearlessly could be used with immunotherapies for best outcomes.

## Introduction

The host body is a city, where the immune system is the fortress guarded by immune “vigilante” components such as lymphocytes, macrophages, mast cells, dendritic cells (DCs), and natural killer (NK) cells. These vigilantes of the immune system always stay alert to keep their fortress safe from the unwanted rioters. Despite this, sometimes cancer emerges as violence due to the internal riot of some cells, called tumor cells. There are six fundamentals of such cells, i.e., growth signal self-sufficiency, insensitivity to signals that inhibit growth, ability to evade apoptosis, ability to sustain angiogenesis, ability to invade surrounding tissues, and metastasis (1). Thereby, the immune vigilantes survey the fort every time and eventually eliminate the unwanted rioters at the very beginning. Sometimes, the rioters come under mutual understanding to stay peacefully into the fort maintaining the equilibrium. But again, some aggressive rioters declined the mutual agreement and cleverly escape the surveillance to build up their unethical agenda and break the citadel gradually. The rioter tumor cells developed several genetic and epigenetic changes to camouflage, which misguide the vigilantes and caused immune evasion (2).

However, if the riot breaks out, the three phases of immune-editing—elimination, equilibrium, and escape (3)—have been strategically modified into different therapies to suppress the riot, such as surgery, hormonal therapy, chemotherapy, radiation therapy, and immunotherapy (4). Hence, in the midst of the personalized therapeutic struggling, cancer immunotherapy has emerged as a new strategy with immense success rates to ban the riot (5). Although it seems very hard to have a full-proof strategy to eliminate the rioters, and even if there is one, the rioters are also prepared with a “counter strategy” to null the efforts of the natives. So, it is much desired to have some booster backup to overpower the rioters. It is indeed a “do or die” situation between our “vigilante” immune system and “rioter” tumor cells.

So, as a “booster back-up” or “an alliance of vigilantes”, curcumin has been taken under consideration due to its cancer combating ability to fulfill the purpose. Since curcumin has the potentiality to suppress tumor cell proliferation by inducing apoptosis, inhibiting angiogenesis, and suppressing the expression of anti-apoptotic proteins; it definitely can be applied as an additional therapeutic booster along with conventional immunotherapies (6–8). Thus, here we are going to discuss the escaping of “rioter” tumor cells, immunotherapies developed to invalidate the riots and the drawbacks of such therapies along with the counter-attack or backup support of the “alliance” curcumin.

## Anti-Cancer Immunity and Immune Evasion Mechanism: The Opposite Doings of Immune-Vigilantes and Rioters

**Figure 1:**
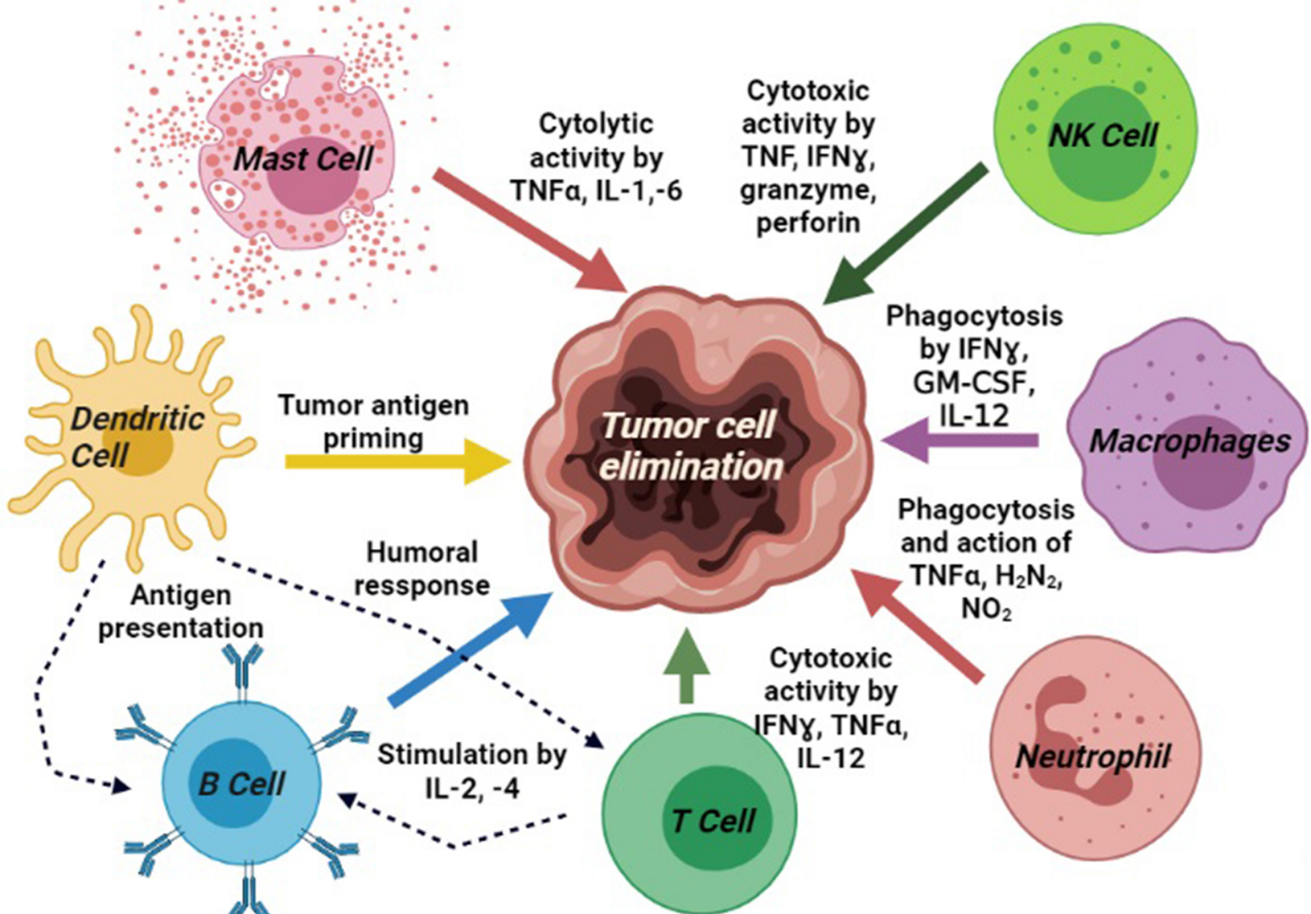
Different immune components portraying the basic signaling pathway modulators and their involvements in cancer immunosurveillance process: tumor antigen priming by DCs; cytotoxic activity by mast cell, T cell, and NK cells; phagocytosis by macrophages; upregulation of neutrophils activity; and induction of humoral response by B cells.

All the molecular blood components, i.e., DCs, NK cells, macrophages, plasma cells, cytokines, antibodies, and helper T cells work altogether for cancer immunosurveillance (9). The NK cells and CD8^+^ T cells mediated cytotoxicity, mast cells mediated cytolysis, phagocytosis by M1 macrophages, and humoral responses by B cells generate anti-tumor activities in the tumor microenvironment (TME). It has been highly expected that tumor-associated antigens (TAAs) produced by early-stage malignant cells will be identified and eliminated by the anticancer immune system (10). DCs are involved in tumor-antigen priming and represent TAAs conjugated with class-I human leukocyte antigens (HLA) to activate CD4^+^ T helper cells and B cells. These reactions are harmonized by several other molecules secreted by the immune cells directly to the TME or into circulation to recruit additional immune populations to the tumor site. CD4^+^ T helper-1 (Th1) boosts T-cell activation, CTL toxicity, and anti-tumor activity of macrophages and NK cells *via* the production of pro-inflammatory cytokines. Besides, CD8^+^ T, which differentiate into cytotoxic T lymphocytes (CTLs), plays the key role as anti-tumor cells (11, 12). CTLs produce the cytokine TNF-α, which has both pro-and anti-tumor properties. It promotes TGFβ release in TME and consequently induces apoptosis in tumor cells (13). IFN-γ is another important pro-inflammatory cytokine, which releases from CTLs and Th1 cells and helps to differentiate CTLs from effector CTLs. It also inhibits angiogenesis in tumor cells, induces the anti-metastatic activities of IL-12, and promotes adaptive immunity (14) (**Figure 1**, created by BioRender).

**Figure 2:**
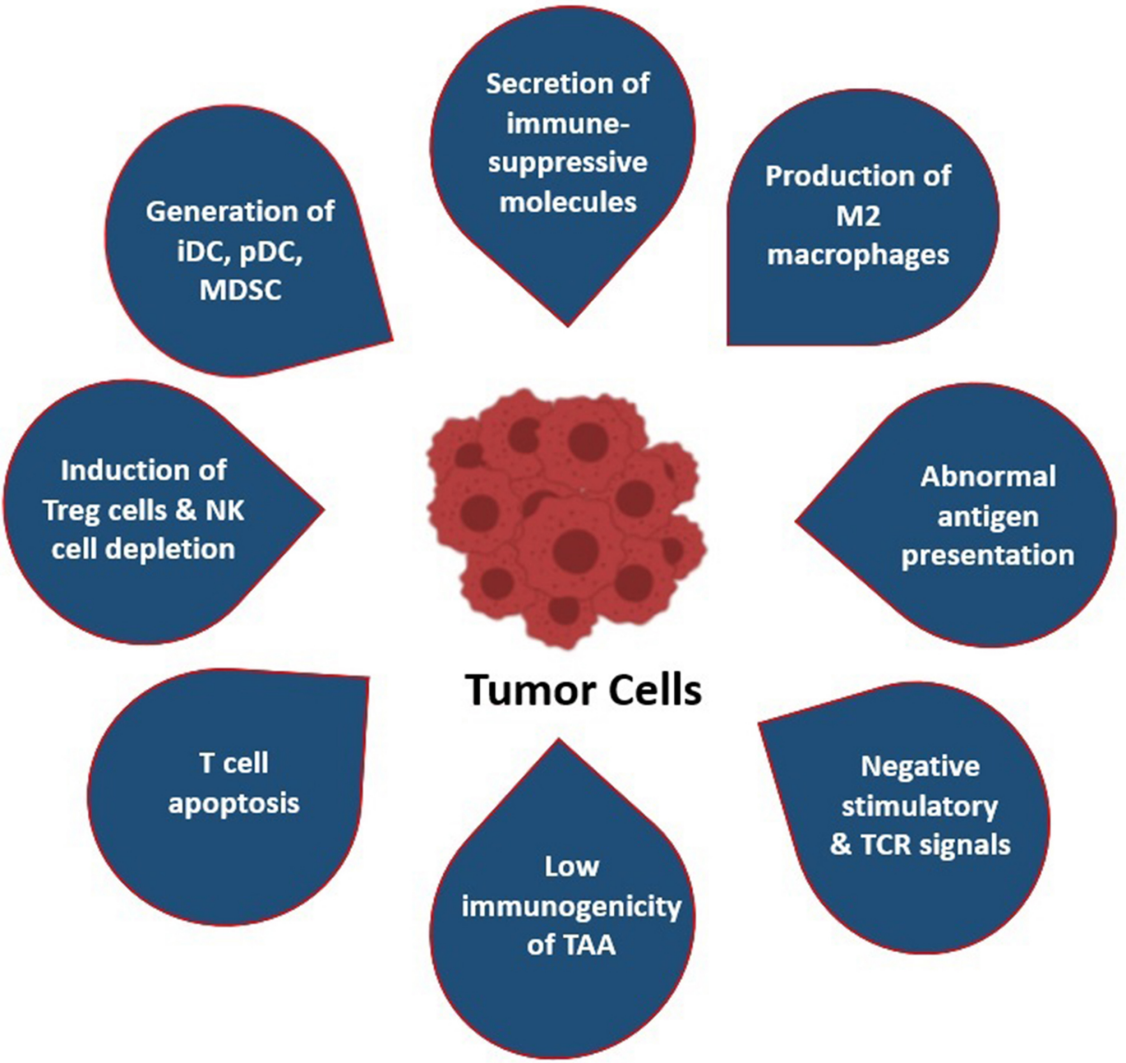
Most common immune evasion strategies by tumor cells: (i) Secretion of immune-suppressor molecules (i.e., CTLA4 and PD1/PDL1); (ii) production and polarization of M2 macrophages; (iii) abnormal antigen presentation by MHC-I and MHC-II molecules; (iv) negative stimulation of TCR signaling pathway by downregulation of antigenicity and upregulation of anergic TME; (v) low immunogenicity of TAA; (vi) T-cell apoptosis and induction of CTL death; (vii) attraction and induction of Tregs and depletion of NK cells; (viii) attraction and polarization of MDSCs, immature DCs, and precursor DC.

In spite of these mechanisms, escaping off the immunosurveillance processes has occurred in TME. Tumor cells themselves generate counter-strategies to overpower the host’s immune system. Numerous studies have reported that tumor cells affect anti-tumor T cells by reducing the infiltration capability of functioning T cells into the tumors, as well as their proliferation, survival, and cytotoxicity (15). The key step of the immune escaping strategy of tumor cells is the conversion of the immunogenic TME into tolerogenic nature (16). Along with this, the surviving tumor cells creates an immune-resistance phenotype, such as it can decrease the release of IFN-γ and induces T-cell exhaustion by producing several immune escape mediators and many signaling pathway modulators, such as stromal barrier/TME, Treg cells, immune-checkpoint inhibitors, respectively (17) (**Figure 2**).

### Immunogenicity to Tolerogenicity

In the TME, tumor cells implement numerous tactics to dodge the immune system and, therefore, establish a tolerogenic environment. It is already established that tumor TAAs are not necessarily neo-antigens that are specifically expressed in tumor cells; rather, they are tissue differentiation antigens that are also expressed in certain healthy cells and create an issue to generate an immune response against such tumor antigens (18). During the late metastasis phase, efficient TAAs release generates effective immunity, but an immune tolerance has been already developed to TAAs by the time and antagonized the function of APCs and other effector T cells (19). Tumor cells also sidestep T cell-mediated immune response by damaging antigen-presenting machinery. Numerous genetic tumor variants by high-frequency mutation result in an escape from immune attack until some antigens are presented by stromal cells and cross-reacted with CTLs for the elimination (20). A large number of tolerogenic cells like Tregs, tumor-associated macrophage (TAM), and tolerogenic DC have been found in the TME. These tolerogenic immune cells release some immunomodulatory molecules, such as TGFβ, IL-10, and PEG2, which foster a tolerogenic environment and block effector immune responses against the tumor growth (16).

In general, activated DCs generate effective co-stimulatory signals to T cells by secreting effector cytokines that stimulate T cells, further leading to tumor cell extinction (21). However, in the TME, tumor cells release various immunosuppressive cytokines that alter immunogenic DCs to tolerogenic. In addition, depletion of toll-like receptor-9 (TLR9) and reduced IFNα secretion by pDCs have been recorded in the TME. This phenomenon aids tumor antigens to escape immune surveillance (22). Macrophages and other APCs play a very malicious role both in the case of tumor growth and regression simultaneously. M1 macrophages exhibit a tumor-suppressive role with killing property, whereas M2 macrophages exhibit an immune-suppressive role with healing phenotype. Tumor-associated macrophages (TAMs) are mainly of M2 phenotypes. They gather in tumor sites and secrete growth-promoting factors like VEGF and EGF. Macrophages also release TGFβ and IL-10, which cause the alteration of effector T cells into Treg cells and also induce immune tolerance (23). Myeloid-derived suppressor cells or MDSCs have the tendency to accumulate around the tumor site and cause severe immunosuppression. There are two general types of pathological MDSCs: monocytic MDSCs and polymorphonuclear MDSCs. In the absence of tumor-derived factors, the monocytic MDSCs and polymorphonuclear MDSCs both turn into macrophages, DCs and neutrophils, respectively. However, the presence of tumor-derived soluble factors induces the formation of immunosuppressive macrophages, TAM, and tolerogenic DC, respectively (24).

### Stromal Cells Barrier on Rioter’s Side

During cancer development, the stromal cells play a “bystander” role. Its interaction with cancer cells decides its competition or cooperation and, accordingly, the suppression and promotion of further tumor progression. It is a thick stromal layer surrounding the malignant tissues and composed of non-malignant cellular and non-cellular connective tissues (25, 26). The components of stroma are extracellular matrix (ECM), fibroblasts, mesenchymal stromal cells, osteoblasts, and chondrocytes (27). These interactions have been involved in the alteration of the genotype and phenotype of cancer cells and create physical barriers with hypoxic condition and abnormal vascularization, which promote tumor growth (28). While potential immune cells are prevented to penetrate the tumor mass, the growing blood vessels help in metastasis, and the efficient release of TAAs in draining lymph nodes is also prevented by stromal cells (9). These stromal layers have a deep impact on tumor therapy. It acts as a tough barrier for therapeutic agents by limiting their access to the target tissues and by degrading the drugs by stromal enzymes (29). Chemo- and radiotherapy induce DNA damage in TME, which raises stress response in stromal cells. This stress response accelerates the secretion of biomolecules, which promote cancer cell survival, proliferation, and metastasis (30, 31). Stromal cells make cancer cell immunotherapy resistant by affecting the concentration of tumor-specific T and B cells. Stromal cells also express immune-checkpoint protein PDL1, but its mechanism on immunotherapy is still ambiguous (32).

### Regulatory Lymphocytes for Misleading

In general, T-regulatory (Tregs) cells are the alternate defense mechanism against autoimmunity, where our immune system fails to eliminate autoreactive T cells without destroying them (33). Surprisingly, Tregs are found in TME, and their survival is promoted by the tumor cells. These are mainly the CD4^+^CD25^+^ immune-suppressive T cells, which suppress the functioning of T cells (34). Tumors are using Treg populations for their own defense against immune surveillance (35). Cancer cells use Treg cells to suppress the immune system by the inhibition of the function of CD4^+^ and CD8^+^T cells, B cells, DCs, and macrophages (36). Likewise, Treg cells can suppress the generation of effector immune cells and produce several immunosuppressant molecules, such as ROS, IL-1, TGFβ, VEGF, prostaglandin E2, adenosine, and galectin 1, and also inhibit antibody production and co-stimulatory molecule expression (37, 38). To search the reasoning behind this behavior, it has been seen that there are two populations of CD4^+^CD25^+^ Tregs, natural and adaptive. The natural population of Tregs originates in the thymus as a defense against autoimmunity, whereas the adaptive Tregs generate during inflammation due to an infection or cancer as a host immunity suppressor (39). Tregs population found in the TME are mostly adaptive but it is not clear yet whether they have differentiated from the natural population or originated from other precursor cells (40).

Like Treg cells, tumor cells also form an inflammatory milieu that induces B-regulatory cell (Breg) population. Immunosuppressive Breg cells suppress immunopathology and block the production of effector T cells and other pro-inflammatory lymphocytes by producing IL-10, IL-35, and TGFβ. IL-10 and TGFβ have the potentiality to convert CD4^+^T cells into Tregs that further would help tumor progression. Breg cells also induce the apoptosis of CD4^+^T cells and the anergy in CD8^+^T cells. By different reports, it has been found that Bregs promote tumor progression by downgrading the production of IFN-γ and IL-17 by Th1 and Th17cells, respectively (41).

### Immune-Checkpoint Receptors as an Alliance of Rioter Tumor Cells

The high mutagenicity and extreme survival capabilities of cancer cells help them to activate several other immune evasion mechanisms in response to their unstoppable growth (42). Upregulation of the checkpoint receptor ligands against the checkpoints PD1 and CTLA4, which downregulate the proliferation and survival of T cells, is considered a major immune evasion mechanism of cancer cells. This phenomenon prevents tumor-infiltrating lymphocytes (TIL) from entering the tumor mass (43). CTLA4, a homolog of CD28, prevents the binding of CD28:B7 after certain stimulatory signaling to check hyperreactivity. The twist of the story is that tumor cells use the mechanism and block the normal binding of CD28:B7 and produces negative signaling. This phenomenon silences the T-cell activation signaling by stopping the IL-2 production and downregulating the cell survival proteins (44, 45). PD1 is another co-stimulatory checkpoint receptor and member of the B7/CD28 family, mainly expressed on activated T cells, B cells, and myeloid cells. It regulates T-cell activation by binding to the B7 family ligands PDLs. PD1:PDLs binding is instigated by the exposure of IFN-γ or tumorigenic signals and generates a negative feedback mechanism to inhibit the immune response. Due to the spontaneous binding of PD1:PDLs, PD1 generates signals to prevent phosphorylation of TCR signaling and inhibits T-cell activation. It also prevents the production of IFN-γ, TNF-α, and IL-2 and reduces T-cell survival (46, 47). In some studies, it has been reported that PD1:PDL1 binding is also responsible for the conversion of naïve CD4+T cells to Treg cells, which further inhibit the anti-tumor T-cell responses (48).

## Immunomodulatory Role of the “Alliance” Curcumin

Turmeric, a common spice obtained from *Curcuma longa* of the Zingiberacea (Ginger) plant family, is the natural source of curcuminoids, a mixture of three different components, i.e., curcumin, demethoxycurcumin, and bisdemethoxycurcumin. A major fraction of this compound mixture is curcumin or diferuloylmethane with 368.38 molecular weight. The crystalline orange-yellow powder is an active polyphenolic phytochemical and has been widely used in medicinal purposes for centuries in India and South Asia, due to its nontoxic but miraculous properties such as anti-oxidant, analgesic, antiseptic, anti-inflammatory, and anti-cancer activity (49, 50). Curcumin as an immunomodulator interacts not just with various cellular components, such as DCs, macrophages, natural killer cells, and both B and T lymphocytes, but also with modulatory molecules involved in the processes of inflammation and cell proliferation with their downstream signaling (51).

**Figure 3:**
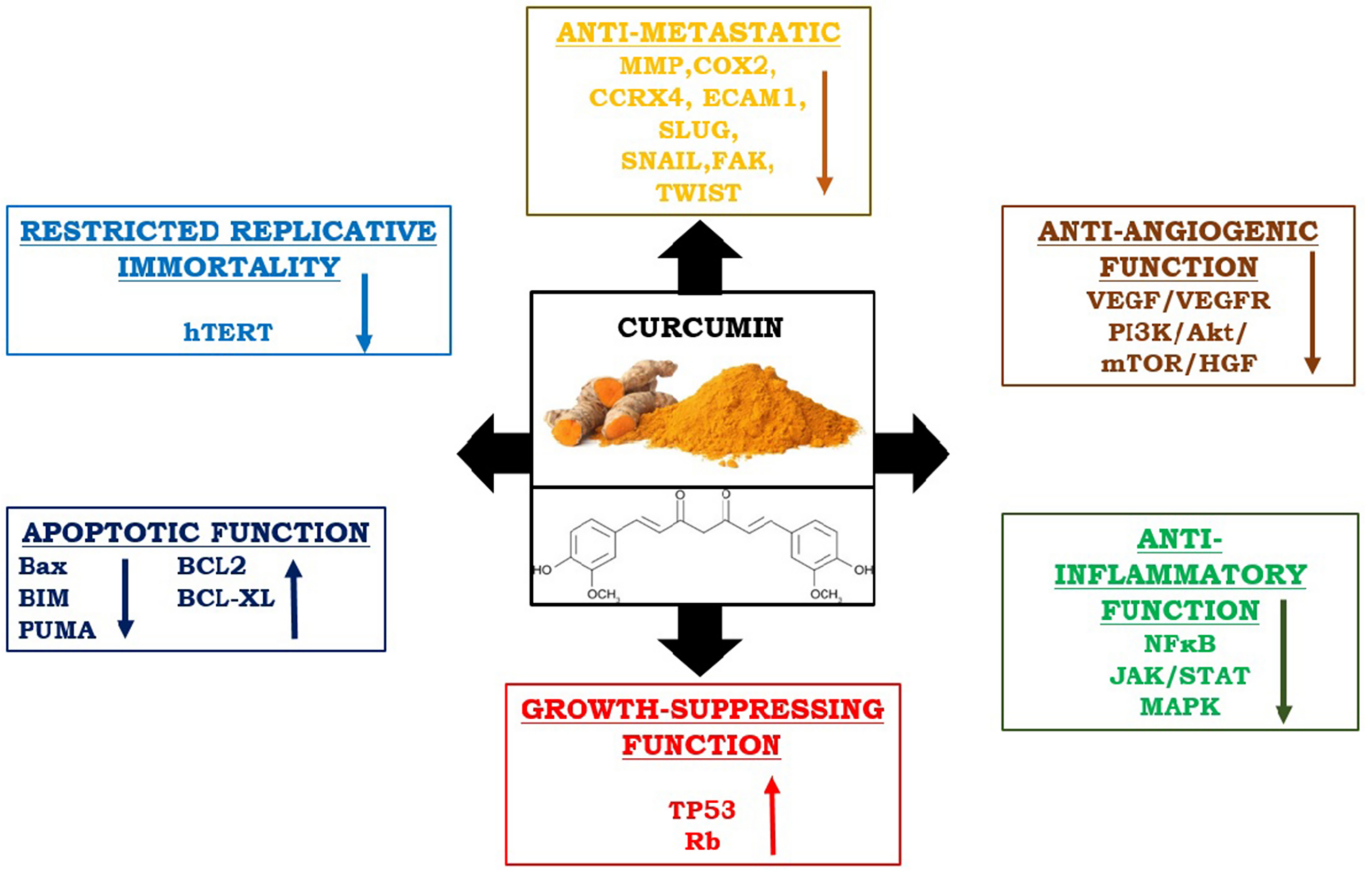
Immunomodulatory and anti-cancer properties of curcumin: It reduces metastasis, angiogenesis, inflammation, and uncontrolled replication by interfering all responsible pathways. On the other hand, it upregulates apoptotic functions by stimulating pro-apoptotic molecules and enhances the function of growth suppressor proteins.

In recent times, curcumin has gained the potential therapeutic interest to cure neoplastic disease, because of its significance as an anti-inflammatory and anti-proliferative substance. The anti-cancer properties of curcumin also modulate several other signaling pathways involved in mutagenesis, oncogene expression, cell cycle regulation, apoptosis, angiogenesis, and metastasis (52). The effectiveness of curcumin has been proven in the restoration of CD4+ and CD8+ cells in the TME and in directing Th2 cytokine bias towards Th1-type response again (53). It increases Th1-type immune responses and upregulates IFN-γ mRNA expression (54). Curcumin effectively reduces Treg cell population and levels of IL-10 and TGFβ. It also can reduce the expression of CTLA4 and FOXP3 both at protein and mRNA levels (55). Interestingly, curcumin has the potentiality to encounter all “six hallmarks” of cancer cells and checks tumor outgrowth in the host (1). Hence, it is considered very interesting to envision the role of curcumin concerning cancer immunotherapies as an immunomodulator (**Figure 3**).

### Effects of Curcumin on Immune Cells

On T cell: Curcumin has the potentiality to modulate the proliferation and activation of T cells. Depending on the dose, it can both suppress and induce the proliferation of T cells. Several studies reported that curcumin downregulates the proliferation of T cells induced by concanavalin A (Con A), phytohemagglutinin (PHA), and phorbol-12-myristate-13-acetate (PMA) (56). Tomita et al. reported that curcumin can suppress the proliferation of HTLV-1-infected T cells and primary ATL cells through cell cycle arrest and induction of apoptosis (57). Research carried out by Hussain et al. stated that in T cell acute lymphoblastic leukemia, curcumin blocks constitutively activated targets of PI3-kinase (AKT, FOXO, and GSK3) in T cells, which lead to the inhibition of proliferation and induction of caspase-dependent apoptosis (58).

On B cell: Curcumin prohibits the proliferation of B-cell lymphoma cells *via* downregulation of c-MYC, BCL-XL, and NFκB activities (59). It also blocks Epstein–Barr virus (EBV)-induced immortalization of B-cells (60).

On macrophage: Curcumin modulates macrophage activities, prevents generation of ROS in macrophages, and stimulates enhanced phagocytosis of peritoneal macrophages in mice (61).

On Natural Killer cell: Curcumin works against natural killer T cell lymphoma cell lines, where it induces apoptosis by controlling the NFκB pathway and suppression of BCLXL, Cyclin D1, etc. (62).

On DC: Curcumin can reduce the expression of CD80, CD86, and class-II antigens by DCs. Curcumin suppresses the release of inflammatory cytokines like IL-1β, IL-6, and TNF-α from LPS-stimulated DCs. Curcumin also modulates phosphorylation of MAPK and nuclear translocation of NFκB in DCs (63).

### Curcumin as an Anti-Inflammatory Substance

It has been observed that chronic inflammation is responsible for several diseases, such as tumor progression, autoimmunity, allergies, and arthritic syndromes. Numerous researches revealed that curcumin can decrease pro-inflammatory cytokines such as IFN-γ, TNF-α, IL-1, and IL-8 by interfering with several signaling and transcription factors such as NF-кB, JAKs/STATs, and MAPK pathways (64).

The anti-inflammatory activity of curcumin mainly depends on its potentiality to inhibit NF-kβ activation. Curcumin inhibits inflammation by downregulating cytokines, IL-1, IL-8, and TNF-α. Curcumin blocks TNF-mediated NF-кB activation in human myeloid ML-1a cells by suppressing activator proteins. Curcumin also blocks NF-кB activation by hydrogen peroxide and phorbol esters. IL-1β-mediated ICAM-1 and IL-8 gene expression are also inhibited by curcumin, which finally leads to the inhibition of NF-кB activation (65, 66).

JAK/STAT is an important signaling pathway in maintaining inflammation in immune cells. It transduces signal type 1 and 2 cytokine receptors in response to pro-inflammatory cytokines. Curcumin inhibits JAK/STAT pathway by blocking the phosphorylation of JAK-1 and -2 and STAT-1 and -2 in IFN-γ, gangliosides, and LPS-activated microglial cells (67, 68).

Curcumin has a distinct role in the inflammatory MAPK pathway. Curcumin significantly lowers the PGE2 (prostaglandin E2) level and the expression of TNF-α and IL-6 by preventing phosphorylation and activation of p38 MAPK functioning (69). Curcumin can suppress LPS-induced phosphorylation of p38, JNK, and ERK1/2-mediated MAPKs pathways and subsequently inhibit the ROS production by microglial cells (70). Kim et al. validated that if immature DCs cells pre-treated with curcumin, it blocked the LPS-induced maturation function of DCs by preventing phosphorylation of p38-, JNK-, and ERK1-/2-mediated MAPK signaling, which consequently checks the inflammation occurrence (63).

### Curcumin as an Anti-Proliferative and Anti-Metastatic Substance

Curcumin acts upon numerous cell proliferation signaling pathways that are intensely associated with cancer progression. Curcumin inhibits NF-кB signaling by suppressing IкB kinase activity. Curcumin suppresses the other proliferation signaling pathways, such as PI3K, AKT, mTOR, AP1 (JUN and FOS), JNK, JAK/STAT, PKC, CMYC, MAPK, ELK, CDKs, iNOS, and Wnt/β-catenin, which confirmed its vital role in the prevention of cancer progression. Cyclin D1, the proto-oncogene that is highly expressed in several types of cancer and acts in cell cycle progression and proliferation, is also suppressed by curcumin (49). Along with this, curcumin also inhibits excessive TGFβ receptor signaling and EGF- and EGFR-mediated signaling pathway and remarkably controls epithelial-to-mesenchymal transition, metastasis, and tumor progression, respectively (71).

A significant activity of the telomerase enzyme has been observed in cancer cells, which prevents telomere shortening and stimulates continuous cell proliferation signaling. Curcumin prevents human telomerase (hTERT) activities and reduces hTERT-mRNA expression that led to telomere shortening. By targeting telomerase activities, controlling replicative cell senescence and mortality, curcumin ultimately controls the uncontrolled cell proliferation of cancer cells (72).

Numerous studies have reported the incredible potentiality of curcumin to inhibit cell migration, invasion, and colony formation *in vitro* and decrease tumor growth and metastasis *in vivo*. Curcumin downgrades the expression of matrix metalloprotease, CCRX4, COX2, ELAM1, and ECAM1, which are essential for metastasis (73). Besides, curcumin also hampers the functioning of SLUG, SNAIL, FAK, TWIST, and other essential transcription factors that play a crucial role in the metastasis process (74).

### Curcumin as an Apoptotic and Anti-Angiogenic Substance

Several studies have reported that curcumin upregulates the expression of p53, which was suppressed by cancer cells and enhances apoptosis (75). Curcumin blocks the phosphorylation of another tumor suppressor protein, RB (Retinoblastoma), which plays a significant role in the cell cycle process (76). Curcumin induces both TP53-dependent and -independent apoptosis of cancer cells by upregulating pro-apoptotic molecules such as BAX, BIM, and PUMA and by downregulating anti-apoptotic molecules like BCL2, BCL-XL, and Survivin. Consequently, the caspase activity gets enhanced and proceeds to apoptosis (77). Besides, curcumin stimulates lysosomal proteases, phosphatases, and lipase activities, which induce autophagy-mediated cell death (78).

Blocking the angiogenesis process is a vital step to control tumor outgrowth. Curcumin suppresses VEGF receptor (VEGFR1 and VEGFR2) expression, blocks VEGF/VEGFR-mediated signaling pathway, and downregulates angiopoietin expression to confine angiogenesis (75).

## Different Types of Immunotherapies and Reasons of Varied Responses: Countered By “Alliance” Curcumin

Immunotherapy is developed depending on seven basic steps, i.e., uptake and processing of the tumor antigen by antigen-presenting cells (APCs), migration of APCs to the lymphoid organs, activation of tumor-specific naïve T cells to effector T cells, trafficking of the tumor tissue by effector T cells, tumor antigen recognition, lysis of the tumor cells, and generation of the tumor-specific memory T cells (79). Based on these cellular and molecular mechanisms, different types of immunotherapies were designed to boost the immune system. Necessary modulation of the regulatory mechanisms has been incorporated with these steps to improve the quality and quantity of the anti-tumor immune responses. There are two categories of cancer immunotherapy: active and passive. Sometimes, these two are combined as an additional option. In the case of active therapy, the host immune system directly attacks the TAAs on the tumor surface, which could be specific proteins or carbohydrates expressed especially in tumor cells with the administrating agents such as mAbs, cytokines, or lymphocytes. On the other hand, passive immunotherapy enhances the basic anticancer response by boosting host immunity (80). This tries to activate the self-immune system for attacking tumor cells through vaccination, nonspecific immunomodulation, or targeting certain antigen receptors. However, the number of responders and non-responders varies widely with the same malignancy after the same immunotherapeutic treatment (81). The inability of immunotherapy to eliminate grown tumors completely is a constant challenge. The numerous cunning suppressive mechanisms of cancer cells and some restrictions of the ongoing immunotherapies are counts as the reasoning behind the failure or wavering responses (35).

As stated before, tumor growth is associated with the escape of immunosurveillance processes and causes general immunosuppression in the body. The manifestations of the phenomenon, such as lower percentages of effector T cells (CD4^+^ and CD8^+^), shifting from Th1- to Th2-type cytokine production, decreased activity of CTLs, and high concentration of Treg cells (82), could possibly be cured by curcumin. Sa and co-workers reported that curcumin is effective in restoring the populations of CD4^+^ and CD8^+^ T cells in the TME and so shifting of the Th2 cytokine towards a Th1-type again (53, 83). Due to the restoration of the Th1 immune response, curcumin is also able to upregulate IFN-γ mRNA expression and promotes cancer regression (54). Bhattacharyya et al. showed that curcumin can effectively reduce Treg population and levels of IL-10 and TGFß (7). It also can reduce the expression of CTLA4 and FOXP3 both at protein and mRNA levels (84).

**Figure 4:**
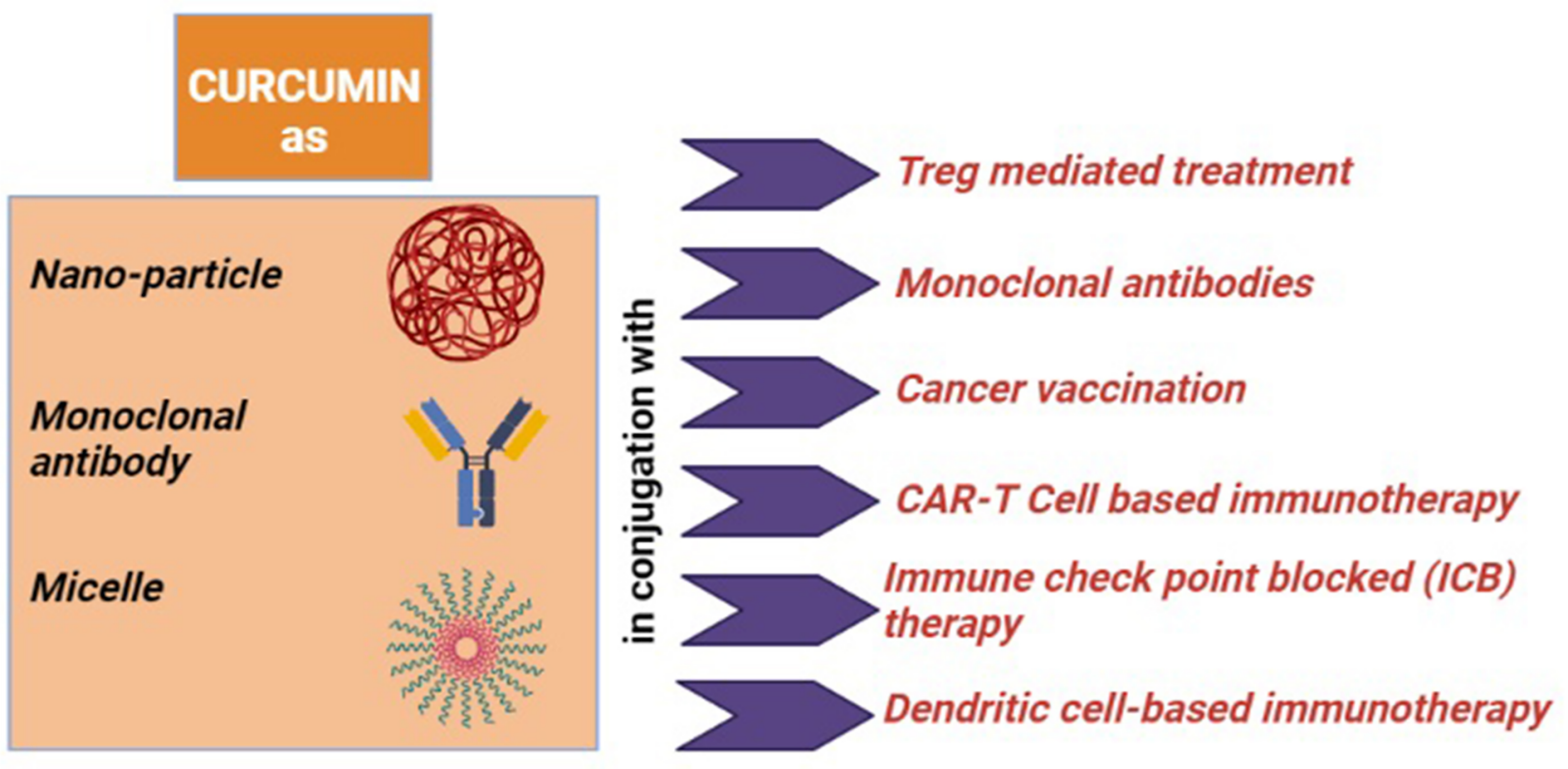
Role of curcumin as an immune-modulator: (i) for the Treg mediated treatment, it converts Tregs into Th1 cell, downregulates FOXP3 and upregulates IFN-γ and CD8^+^ T cells; (ii) when it conjugated with monoclonal antibody (mAb), it downregulates NF-кB and tumor-promoting proteins; (iii) when co-associated with cancer vaccination, it reduces immunosuppressive factors and IL-6 and induces TNF-α, IFN-γ, and CD8^+^ T cells; (iv) curcumin with adoptive T-cell therapy (Act and CAR-T cell) induces and activates CD8^+^ T cells; (v) as an immune-modulator with immune checkpoint blockade (ICB) therapy, it reduces Tregs, CTLA4, and PDL1 altogether; (vi) it inhibits STAT3 signaling and IL-6 and IL-8 levels, when applied with DC-based immunotherapy.

In the next part, we will discuss the conventional immunotherapies that are applied against tumor immune evasion and the role of curcumin as an immune modulator to recreate tumor immune surveillance from tumor immune escape (**Figure 4**, created by BioRender).

### Treg-Mediated Treatment and Its Ambiguity

Progression of tumor is associated with Treg cell population augmentation in the TME and correlates with poor prognosis of cancer (85). It not only secretes immunosuppressive cytokines like TGFβ and IL-10 but also expresses high-affinity IL-2 receptor CD25, which sequesters IL-2 from the tumor milieu. Subsequently, it leads to the effector T-cell apoptosis (86). In the scenario, Tregs are a good target for immunotherapy in cancer patients (39). The goal of this therapy is to downregulate the Treg population to increase the level of IL-2 and increase the survivability of effector T cells as well. Few FDA-approved drugs have been used to treat mostly CD4^+^CD25^+^Treg cells (87). Denileukin Diftitox, a recombinant drug combining IL-2 and diphtheria toxin, has been frequently used for leukemia therapy and also for another carcinoma. A phase 1 clinical trial with advanced stage 3 epithelial carcinoma has been conducted and a positive response has been observed with a dose of 9 or 12 μg/kg Denileukin Diftitox (88). Despite this, failure has been observed with cancer-induced antigen-specific Tregs (89). The identification of individual functional Tregs is still a major concern as only a subset of T cells are found to be CD4^+^CD25^+^ by flow cytometry, which is eventually suppressive Tregs in nature (33). Studies reported that transcription factor fork-head box P3 (FOXP3) expression is crucial for the development of the CD4^+^CD25^+^ Tregs. Interestingly, not all FOXP3^+^ T cells are suppressive Tregs; instead, some FOXP3^-^ T cells are suppressive in nature as well (35). Further works are needed to identify the functional Tregs with a very appropriate marker as a definite sign of CD4^+^CD25^+^ Tregs (90). Another important issue is why the number of Tregs is increased in cancer patients. Some questions arise: Are the cells trying to suppress anti-tumor immunity recognized as an auto-reactive immune response? Are they reacting to the inflammation? Is there some other issue? These unanswered questions make the therapy difficult and bring forth additional confusion, such as which local factor in the TME acts as a catalyst for Tregs, what is the precursor population of Tregs, what is antigen specificity of Tregs, and how it is affecting effector immune cells. Practical practice in therapeutic background highlighted the fact that the agents used to reduce Tregs function can work correspondingly well or not in all kinds of mediums, such as blood, tumor, and draining lymph nodes. The relationship between Treg depletion in the blood and at local sites is still ambiguous, and whether such observations are effective to predict immunologic or clinical outcomes remains unknown (35).

In context to overcome these ambiguities, several studies have indicated that curcumin could target Treg cells and convert them into Th1 cells, which possess anti-tumor effects. The results showed that after 2 weeks of treatment with curcumin, the number of peripheral Treg cells distinctly declined while the number of peripheral Th1 cells was improved. It has been assumed that curcumin enhanced the conversion of Treg cells to Th1 cells by suppressing the expression of Foxp3 and inducing the expression of IFN-γ (91). A recent *in vitro* study showed that curcumin could inhibit the suppressive activity of CD4^+^CD25^+^Treg cells *via* inhibition of cytokine IL-2 secretion as well as *via* reduction of Foxp3 expression in Treg cells (55). Furthermore, after treatment with curcumin, an enhancement of CD8^+^T cells has been registered instead of Treg cells, which resulted in a boost in anti-tumor immune responses (92). On this ground, it is evident that curcumin has the potentiality in suppressing the activity of Treg cells, and due to its significant role, it may be possible to use curcumin as an immunotherapy modulator for Treg-mediated therapy in the future.

### Monoclonal Antibodies and Its Swing Response

Monoclonal antibodies (mAbs) are engineered substitution antibodies that can restore, enhance, or mimic the immune system’s attack on cancer cells by targeting the antigens that present enormously on the surface of cancer cells than healthy cells. The Food and Drug Administration (FDA) has approved significant numbers of mAbs to treat solid and hematological malignancies and more novel mAbs are under clinical trials (93). Presently, several types of mAbs have been registered, such as conjugated, bi-specific, and naked mAbs that are applied to treat cancers (94). Conjugated mAbs can be linked to a chemotherapy agent or a radioactive particle, which can carry these materials instantly to the tumor cell (95). Brentuximab vedotin, a chemo-labeled conjugate mAb, is targeting CD30 antigen that presents on lymphocytes, used for the treatment of Hodgkin lymphoma and anaplastic large cell lymphoma (96, 97). Similarly, a radio-labeled antibody has a radio-active particle attached to it (98). Bispecific mAbs consist of two various parts of mAbs, e.g., blinatumomab, bounded to CD3 and CD19 and are applied for treating acute lymphocytic leukemia (ALL) (99). The most common form of mAbs is naked mAbs, which is highly used to treat cancer. However, the clinical success rate of this therapy is still not satisfactory. The prime reason is the lack of specificity of the tumor neo-antigens that can be targeted by mAbs. TAAs are not a specific tumor neo-antigen, rather they are tissue differentiation antigens, which correspondingly expressed in certain normal healthy cells. Additionally, frequent genetic mutations during tumor development generate secondary neo-antigens, which unfortunately escape from primary recognition, as CTLs are only able to identify the primary neo-antigens expressed on HLA class I molecules (100). So, the specificity between CTLs and TAA is a major barrier for this therapy. In some conditions, the developed malignant cells and TME turn off the expressed TAAs through the process of immune tolerance induction and inhibits anti-tumor response, respectively (9). Accordingly, some common side effects, such as low blood pressure, headache, fever, nausea, chill, weakness, and diarrhea, have been recorded, along with several serious health issues, which might be lethal in some cases (101). Last but not the least, the production cost of this therapy is very high.

Though individually this therapy is not found very encouraging, when curcumin is linked with the cleavable arm to a mAb, its functional efficacy has been increased up to 230-fold more in eliminating murine melanoma B16F10 cell-induced brain tumor. Curcumin‐mAb treatment resulted in an inhibition of NF‐kB. Thus, by suppressing NF‐kB, the curcumin-mAb inhibits the downstream tumor‐promoting proteins, thereby eradicating the B16F10 cells. So, such a novel applicable strategy of converting curcumin into a potent anticancer immunomodulatory agent may provide a mechanistic framework for its action (102).

### Cancer Vaccine and Restriction of Its Specificity

Cancer vaccine is the immune modifier, which acts *via* stimulation or re-establishment of the immune system competence to combat cancers (103). It is similar to monoclonal antibody therapy, which involves conventional vaccination methods to induce an immune response. Whole cancer cells or a particular protein or peptide are used to trigger the immune system (104). Two general types of vaccines are mainly applied, i.e., preventive and treatment vaccines (105). Preventive vaccines work depending on the antigens taken by infectious factors so that detection of them by the immune system can be easy (106). It activates the immune system with the targeted T cells for eliminating target tumor cells. The FDA-approved hepatitis B virus (HBV) vaccine and human papilloma virus (HPV) vaccine are such kinds of vaccines (107). Therapeutic vaccines target the immune system directly and boost the immune system’s attack on cancer cells. Besides, its capacity to invade other tumor-specific antigens as well develops immune responses equally (5). However, the main reason for the differences in patient response rate is the antigen specificity of the vaccine as the vaccine is designed based on the peptide antigen complexes with HLA present on tumor cells (100). Thus, designing a commercial form of the vaccine is very much patient- and tumor type-specific and needs to be administrated to a minimal subset of the patient that expresses the same antigen.

Even though the main reason for its failure is lack of specificity, it is really hard to commercialize a standard tumor antigen. Thus, necessary immunomodulation has been considered as a remedy. With an intention to study the immunomodulatory effects of curcumin in cancer treatment, curcumin–polyethylene glycol (PEG) conjugate, an amphiphilic curcumin-based micelle, has been used in combination with conventional vaccine therapy in an advanced melanoma model. This combination resulted in a synergic anti-tumor effect and a depletion in the levels of immunosuppressive factors, such as myeloid-derived suppressor cells and Treg cells. In addition, IL-6 levels were reduced in TME and the concentration of pro-inflammatory cytokines, such as TNF-α, IFN-γ, and CD8^+^T cell population was elevated. Hence, it can be suggested that derivatives of curcumin conjugate may be used as a potential modulator to promote cancer vaccination not only in advanced melanoma cases but also for other carcinomas (108).

### Cell Based Immunotherapy: Adoptive T Cell Therapy and T-Cell Engineering

One of the most common forms of immunotherapy for hematological and solid malignancies is the ACT of TAA-specific T cells (109). Previous researches on the ACTs are based on the use of TILs, though this strategy has been constrained due to the problems for expansion of viable TILs and restricted exhibition of certain effector functions (110). Hence, for resolving the problems, applications based on chimeric antigen receptor (CAR) and TCR-engineered T cells have been developed that act *via* genetic modification and increase the therapeutic efficiency (111). One of the conventional therapies for metastasis melanoma is to transfer antigen-specific TCR genes into lymphocytes by transducing T cells with retroviruses or lentiviruses to ensure the expression of TCRs. This has been applied to target certain TAA and eliminate tumor cells (112). Although, in metastatic melanoma patients, the outcomes seem very promising, in general, TCR technology has been found less fascinating, as it is MHC-restricted (113). CAR-modified T cells are the second group of the engineered T cells, applied to enhance the specificity and anti-tumor capacity (9). In comparison with TCRs, CAR-T cells exhibit antibody-like specifications that could detect MHC-non-restricted structures on the target cell surfaces and subsequently recognize tumor cells in an MHC-un-restricted mode (114). However, this is the main disadvantage of its application. Since there are only a few tumor-specific cell surface antigens, it restricted the specificity of CAR-T cells and narrowed the application (79). Even though CAR-T cells are found to be very effective for the treatment of CD19+B cell lymphomas and leukemia cells, it also destroys all the normal CD19+B cells in the patient body (115, 116). In addition, acute cytokine release syndrome (CRS), as a result of the supraphysiologic level of cytokine production upon antigen recognition, is a common side effect of this therapy. These drawbacks limited its use as a therapeutic option for both solid and hematological tumors (117, 118).

Very little is known about the effect of curcumin when it is combined with T-cell-based therapy, such as ACT or CAR T cells both in *in vitro* and *in vivo* models. Chang et al. exhibited in an *in vitro* study that the activation of CD8+ T cells was significantly increased when adoptive T-cell therapy co-applied with curcumin pre-treated E.G7 tumor cells along with the inhibition of TGFß. This research suggested that the multi-targeting benefit and immunomodulation of curcumin may condense the efficiency of adoptive T-cell therapy in combination treatment (119).

### Immune Checkpoint Block Therapy and Its Side Effects

Immune checkpoints are essential for host immune backup to prevent autoimmunity. It has been observed that cancer cells use this mechanism to escape from the host immunity system by deactivating TILs, concerning the activation of T cells. Immune checkpoint blockers are developed with the aim of increasing immune responses against cancer (120). T-cell surface molecules, CTLA4, PD1, T-cell immunoglobulin and mucin domain 3 (Tim-3), and lymphocyte activation gene 3 (LAG3) are some essential checkpoint modulators (121). Tumor expression of these markers would lead to enervation of the immune system (122). Thus, the above molecules become the center of attention by researchers as the target (123). PD1 and CTLA4 are the two most common checkpoints clinically targeted for treatment. Immune checkpoint inhibitors are mainly the types of mAbs that block immune checkpoint receptors and allow activated T cells to clear tumor cells (9). PD1 or CTLA4 blocking results in deactivation of Tregs and other immunosuppressive mechanisms and consequently activated anti-tumor T cells for defense (10). In recent times, nearly 100 clinical trials are ongoing for testing the efficiency and safety of ICB in different types of cancers (120). Although this therapeutic approach is found to be very effective, a varying response rate with some side effects has been observed among patient cohorts with the same cancer type (81). Specific consequences such as hepatic, gastrointestinal, endocrine, dermatologic, and other less prevalent inflammatory syndromes are highly associated with checkpoint inhibition. Anti-PD1 therapy needs more research on TME to identify the exact mechanism of its upregulation there (79). Clinically, it has been observed that anti-CTLA4 or anti-PD1 mAbs induce autoimmune reactions. Hence, the question arises regarding its control over auto-reactive T cells, and this insufficient understanding of the mechanism restricted its use as a single-agent therapy (124, 125).

The role of curcumin on immune checkpoints is not clear yet and requires more researches. In parallel, some basic anti-tumor functioning of curcumin such as ICB therapy assumes to be effective. CTLA4 is one of the key transcription factors, involved in the regulation of the Treg transcriptional program and is vital for Treg development and functioning (126). The study has shown that curcumin can reduce the expression of CTLA4 both at protein and mRNA levels (55). Curcumin is also effective in reducing the number of Treg cells, which further reduces the number of CTLA4 consecutively. Numerous studies have reported that curcumin has the potentiality to convert Treg cells into Th1 cells, which possess anti-tumor effects (91). Another study reported the effect of curcumin on immune suppression in tongue squamous cell carcinoma. Curcumin treatment inhibited the expression of the PDL1 both *in vivo* and *in vitro*, which sequentially blocked the binding with PD1 and promote anti-tumor T-cell proliferation (38). Hence, curcumin has been shown to modulate the expression of CTLA4 and PDL1 and application of curcumin together with immune checkpoint blockers may establish a better approach to eradicate tumor cells.

### DC-Based Immunotherapy and Curcumin Association

DCs are very distinct and potential antigen-presenting cells. It represents TAAs effectively to generate tumor-specific immunity. Numerous animal studies have confirmed that DCs can stimulate T cell-mediated tumor elimination when loaded *ex vivo* with tumor antigens. These observations have prompted inspecting the immunological and clinical effects of antigen-loaded DCs directed as a therapeutic vaccine to cancer patients. The outcomes from these clinical trials in patients with malignant lymphoma, melanoma, and prostate cancer advocate these immunotherapeutic strategies that would be beneficial with the antigen-presenting properties of DCs and might be proven as efficient and widely applicable to human tumors (127).

Irrespective of these parameters, it has been observed that STAT3 activation in DCs and Tregs in TME involves the suppression of the anti-tumor immune responses. Various cytokines, such as IL-6 and IL-8, play immune-suppressive roles by inhibiting DC function and alternatively by uprising MDSCs and Tregs (128). The uses of curcumin are mainly prescribed in the case of inflammatory or autoimmune diseases as curcumin suppresses the activation of DCs by modulating JAK/STAT/SOCS or MAPK pathways (84). It has been observed that curcumin basically inhibits the immune-stimulatory function of DCs and interferes in myeloid DC maturation (64). Kim et al. reported that curcumin downregulates the production of pro-inflammatory cytokines, such as IL-1, IL-6, and TNF-α, from maturing DCs, and it has been observed that curcumin-treated DCs exhibited suppression of Th1 responses, confirming the inhibitory effect of curcumin on DCs maturation (63). In spite of that, recent studies have reported that in some cases, curcumin indirectly helps DCs to generate tumor-specific immune responses. Curcumin enhances anti-tumor T-cell responses by inhibiting STAT3-activated inflammatory signaling in TME and also in DCs, which reestablishes the anti-tumor response again. It has been demonstrated that curcumin indirectly restores the functioning of DCs by reducing IL-6 and IL-8 productions by cancer cells *via* STAT3 inhibition (129). So, the use of curcumin as an additional modulator with DC-based immunotherapy could be a wise thought but it still requires more research to understand the mechanism.

## Application of “Alliance” Curcumin as an Adjuvant in Cancer Immunotherapy

In order to offer a rational model for achieving optimum efficiency, it is highly essential to understand properly the particular mechanism of the parent medicine and adjuvant combinations (130). Combinatorial impacts have been obtained by combining two specific partner medicines with lesser, equivalent, and or greater impact. Such impacts might be antagonistic, potentiation, and synergistic (131). If the obtained impact is better than the whole impact of the medicines without cross-reactions and several functioning targets or paths, it will be considered as a synergistic combinatorial impact (132). In case the impact is higher than or equivalent to the whole impact of individual medicines with some distinct targets or pathways, it will be the additive impact. When the activity or impact of one medicine can be seen by the other, it would be potentiation impact (133, 134).

Curcumin has multiple potentials due to its numerous antineoplastic mechanisms for cancer therapy. Curcumin, a chemo-sensitizing agent, also enhances the efficacy of several chemotherapeutic agents (131). According to the study by Chen’s group, among four anticancer chemotherapeutic factors (erlotinib, sorafenib, sunitinib, and doxorubicin), sunitinib combined with curcumin at a molar ratio of 0.46 achieved the most potent synergistic effect *in vitro* and has been selected to be studied in an animal model (135). The anti-tumor effects of curcumin or turmeric extract in combination with bevacizumab in HT29 colon tumor-bearing mice have been examined by Yue’s group. When curcumin is combined with bevacizumab therapy, it suppressed tumor growth significantly with no physical side effects (132). This highly indicates the therapeutic promise of adjuvant application of curcumin for treating cancer, particularly combined with several mAbs (102). A clinical trial has been executed by Basak et al. with oral cancer patients where APG-157, a botanical drug containing multiple polyphenols, including curcumin has been administrated orally. According to the study, APG-157 was absorbed well and significant trace of curcumin has been found in the blood and in tumor tissues. This trial reported the downregulation of inflammatory markers and *Bacteroides* species in the saliva and upregulation of the immune T cells in the tumor tissue. Additionally, it reduced inflammation and attracted cytotoxic T cells to the tumor site, signifying its potential usage in combination with immunotherapy drugs (136).

## Limitations on the *In Vivo* Use of Curcumin and the Ways to Overcome Them

Though curcumin has been utilized as a promising agent for chemoprevention and cancer immunotherapy, it is not considered as a cure for everything from a clinical perspective. Since curcumin has poor aqueous solubility, i.e., 11 ng/ml in water, it causes difficulties in oral administration (137). It is hydrolyzed fast and only soluble in acidic conditions (138). After intravenous or intraperitoneal administration, a significant amount of curcumin is excreted *via* bile in the form of hexahydrocurcumin and tetrahydrocurcumin glucuronide derivatives (139). The poor bioavailability of oral curcumin in the GI tract restricts its potentiality against cancer immunosuppression (140). Even after the oral daily intake of 3600 mg or higher doses of curcumin in patients with advanced colorectal cancer, the detected concentration of curcumin in peripheral and portal blood was in nanomolar levels (141). It has been assumed that the main reasons for such poor bioavailability are its poor absorption, rapid metabolism, chemical instability, and rapid systemic elimination. Along with poor aqueous solubility and low bioavailability problem, several other therapeutic issues have emerged over time such as lack of dose–response proportionality, uncontrolled precipitation, use of excessive co-solvents, and requirement of safe medium to solubilize (basic or acidic), which impedes its effectiveness as a chemotherapeutic drug against cancer (142). Thus, the development of an appropriate strategy to upgrade the bioavailability and solubility of curcumin is a vital concern for a beneficial therapeutic approach against immunosuppression induced by tumors.

**Figure 5:**
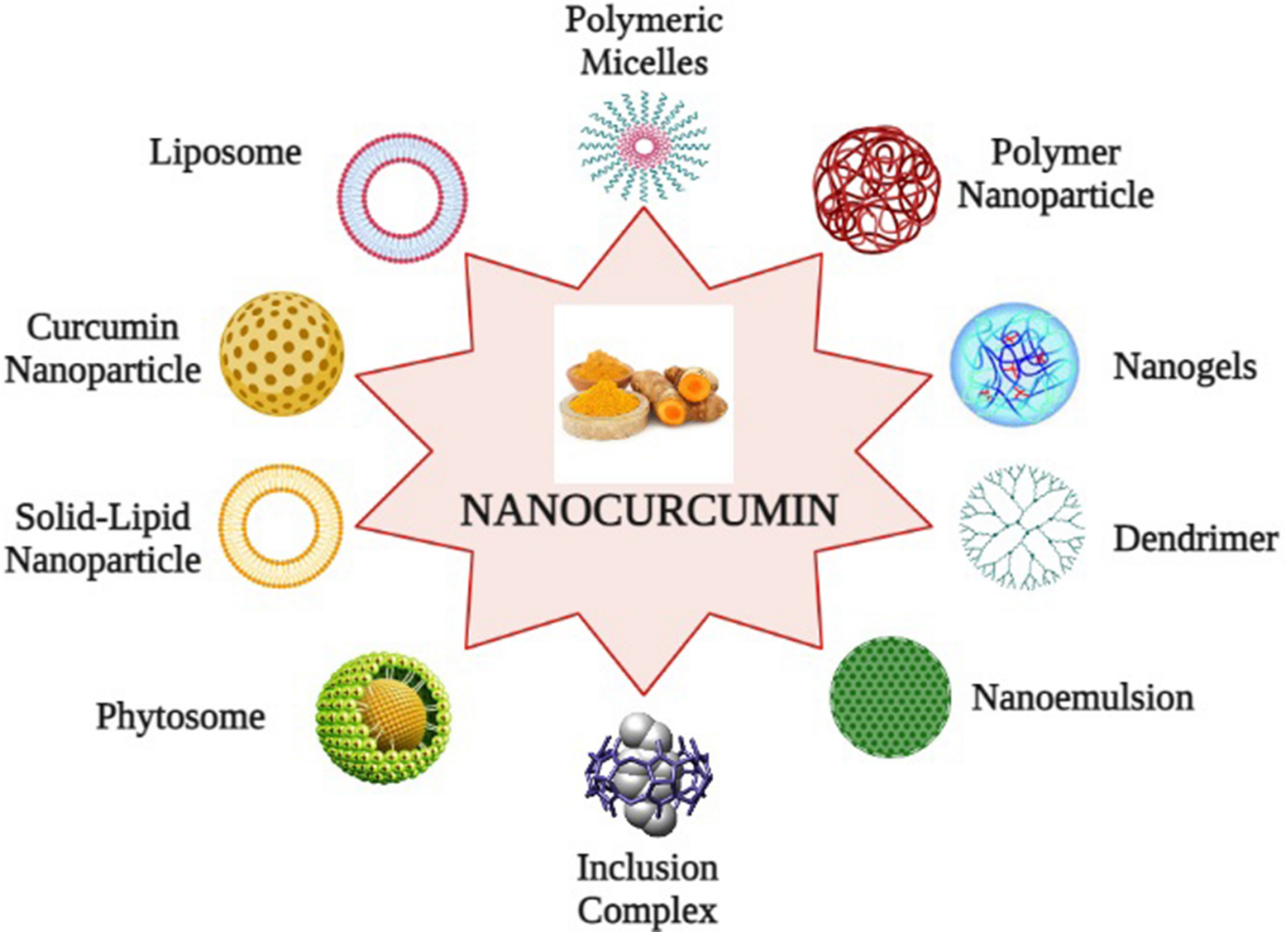
Curcumin nano formulations: (1) Liposomes, (2) Polymeric micelles, (3) Polymer nanoparticles, (4) Nanogels, (5) Nanoemulsion, (6) Solid lipid nanoparticles, (7) Inclusion complex, (8) Dendrimer, (9) Phytosome, and (10) Curcumin nanoparticles.

To overcome these limitations, nanotechnology-based drug delivery systems have been established as a reliable and promising approach. Nanotechnology-based drug delivery systems of curcumin not only improve poor bioavailability but also enhance biological activities and selectively target cancer cells. This technology packs the active pharmaceutical ingredient of curcumin into nano-sized particles, ranging from 10 to 1000 nm to enhance systemic bioavailability. It also enhances the dissolution rate of curcumin and appears as an applicable method to deliver insoluble drugs (143). Numerous nanoparticle formulations are developed for effective encapsulation of curcumin, which includes *Liposomes*: lipophilic spherical particles are incorporated into the hydrocarbon bilayer whereas hydrophilic units are fused into their aqueous interiors; *Nanoparticles:* a particle of 1–100 nm diameter, consisting of a concentrated matrix structure that can be combined with the active ingredient of the drugs; *Micelles*: Micelles are amphiphilic molecules of 20–100 nm diameter and help in better solubilization and targeted delivery to curcumin; *Nanogels*: It is a core shell polystyrene gel layer structure composed of an inner hydrophobic core for the interaction with the active pharmacological substances of the drug and a PEG analogue outer shell for triggering fast release of the preloaded drug; *Nanoemulsions*: Thermodynamically stable dispersion of aqueous and oil substrates, and it is stabilized with active surface film consisting of surfactant and co-transfactant; *Phytosome complexes*: A phospholipid complex, formed by pure phospholipids containing biological derivatives with active pure ingredients with physicochemical and spectroscopic properties; *Inclusion complexes*: It is a mixture of active drug ingredients located in the hydrophobic cavity of bulky host molecules such as cyclodextrin, a bucket-shaped oligosaccharide. It is a solubilizing and stabilizing agent that can solubilize the curcumin in a lipophilic cavity. The outer hydrophilic surface contributes in greater dispersion of the formulation; *Dendrimer or dimers*: It is a core–shell nanostructure, synthesized in a layer-by-layer fashion where several pharmaceutical active compounds are directly connected by stable chemical bonding; and *Solid lipid nanoparticles (SLNs):* SLNs are an aspherical lipid core matrix that can solubilize curcumin and the lipid core is stabilized through emulsifiers (49, 144, 145). The encapsulation of curcumin into the exosomes, the extracellular secretary nano-vesicles, also enhances its stability, solubility, and bioavailability (146). Furthermore, several bio-enhancers are also available such as piperine, quercetin, and silibinin, which prevent or reduce the metabolism of curcumin, and it has been expected that application of these bio-enhancers with curcumin as dual drug-loaded nanoparticles will be advantageous (147). Recently, pure curcumin nanoparticles without any carrier conjugates have been developed, which have 50 times more effectiveness and better bioavailability than normal curcumin and have shown the potentiality to suppress markers of Treg cells and recover immune responses in experimental models. They are developed by dissolving pure curcumin in ethanol and homogenization at high pressure with water containing 0.1% citric acid (148) (**Figure 5**, created by BioRender).

Even though nanotechnology-based drug delivery has a promising future towards effective cancer therapy, there are still certain limitations. Complications regarding drug targeting and loading capacity, *in vivo* fate of the carrier–molecule conjugates, toxic effects of the carrier molecule, its large-scale production, stability during long-term storage, and overall production costs are challenging to deal with. Specifically, the toxic effects of nano-formulations in the body are definitely an emergent concern. Even after the toxicity and biocompatibility of the carrier materials are tested, the properties of the nanoparticles still differ frequently. Hence, before approval for a clinical trial, thorough evaluations are extremely required to determine the toxicity of the carrier materials and their metabolites (142).

## Conclusion

The violation of cancer is one of the leading causes of death worldwide and it is still a challenge to find out some promising therapeutic approaches against the rioter tumor cells. One of the major concerns is the immune evasion of tumor cells from the vigilant host’s immunity. The soldiers of the immune system play major roles in eradicating tumor cells. Hence, boosting up the soldiers with biotechnology and genetic engineering is considered as an advantageous development to fight cancer cells and also develop novel immunotherapies. Despite its immense success rate, some weaknesses are still haunted by its maximum success, which has been already discussed above.

The “alliance” curcumin has distinct therapeutic activities including anti-oxidant, anti-inflammatory, and anti-microbial properties and is thus found to be an extremely promising anti-cancer agent, targeting various immunological pathways associated with cancer evolution. Studies continue to reveal its potentiality to interact with the immune system, emerging as an important booster due to its anti-cancer properties. Since immunotherapies have been developed to eliminate tumor cells overruling their escape mechanisms, curcumin can be used as a potent immunomodulator or booster backup.

Even though several reports have submitted the general immunosuppressive role of curcumin and its low bioavailability in circulation, numerous studies report that curcumin increases anti-tumor immunity through various modes, as discussed in this review. Therefore, modifications of the conventional immunotherapies seem to be an important strategy by which curcumin counteracts cancer development. This further proclaims its effectiveness as an anti-cancer agent and points out the need to use it as an adjuvant immunotherapeutic agent. This leads to the development of nano-based strategies for appropriate delivery and increased bioavailability of curcumin, which may finally lead to its use as a proper immunotherapeutic modulator.

## Author Contributions

SP undertook the background literature study and prepared the initial draft of the manuscript and the figures. GS supervised the entire project and made final corrections to the draft. All authors contributed to the article and approved the submitted version.

## Funding

This work was supported by research grants from the Department of Biotechnology, Government of India. GS is a National Academy of Science (India) Platinum Jubilee Senior Scientist.

## Conflict of Interest

The authors declare that the research was conducted in the absence of any commercial or financial relationships that could be construed as a potential conflict of interest.

## Publisher’s Note

All claims expressed in this article are solely those of the authors and do not necessarily represent those of their affiliated organizations, or those of the publisher, the editors and the reviewers. Any product that may be evaluated in this article, or claim that may be made by its manufacturer, is not guaranteed or endorsed by the publisher.

## Abbreviations

DCs, Dendritic cells; NK cells, Natural killer cells; TME, Tumor microenvironment; TAA, Tumor-associated antigen; CTLs, Cytotoxic T lymphocytes; TNF-α, Tumor necrosis factor alpha; IFN-γ, Interferon gamma; TGFβ, Transforming growth factor beta; IL, Interleukin; GM-CSF, Granulocyte macrophage–colony stimulating factor; APC, Antigen-presenting cell; ROS, Reactive oxygen species; VEGF, Vascular endothelial growth factor; CTLA4, Cytotoxic T-lymphocyte-associated protein-4; PD1, Programmed cell death protein 1; MHC, Major histocompatibility complex; FOXP3, Forkhead Box P3; FOXO, Forkhead Box O; BCL, B-cell lymphoma; MAPK, Mitogen-activated protein kinases; JAK/STAT, Janus kinases/signal transducer and activator of transcription proteins; NF-кB, Nuclear factor kappa light chain enhancer of activated B cells; ICAM, Intercellular adhesion molecule; hTERT, Human telomerase reverse transcriptase; COX2, Cyclooxygenase-2; ECAM1, Extracellular adhesion molecule; FAK, Focal adhesion kinase; TWIST, Twist-related protein ; MDSCs, Myeloid-derived suppressor cells; AKT, Protein kinase B/Ak strain transforming; GSK3, Glycogen synthase kinase 3; LAG3, Lymphocyte activation gene 3.

## References

[1] Hanahan D Weinberg RA. The Hallmarks of Cancer. Cell (2000) 100:57–70. 10.1016/j.cell.2011.02.013 10647931

[2] Shankaran V Ikeda H Bruce AT White JM Swanson PE Old LJ. IFN and Lymphocytes Prevent Primary Tumor Development and Shape Tumor Immunogenicity. Nature (2001) 410:1107–11. 10.1038/35074122 11323675

[3] Dunn GP Bruce AT Ikeda H Old LJ Schreiber RD. Cancer Immunoediting: From Immunosurveillance to Tumor Escape. Nat Immunol (2002) 3:991–8. 10.1038/ni1102-991 12407406

[4] Panda AK Chakraborty D Sarkar I Khan T Sa G. New Insights Into Therapeutic Activity and Anticancer Properties of Curcumin. J Exp Pharmacol (2017) 9:31–45. 10.2147/JEP.S70568 28435333 PMC5386596

[5] Yang Y. Cancer Immunotherapy: Harnessing the Immune Systemto Battle Cancer. J Clin Invest (2015) 125(9):3335–7. 10.1172/JCI83871 PMC4588312 26325031

[6] Choudhuri T Pal S Das T Sa G. Curcumin Selectively Induces Apoptosis in Deregulated Cyclin D1-Expressed Cells at G2 Phase of Cell Cycle in a P53-Dependent Manner. J Biol Chem (2005) 280:20059–68. 10.1074/jbc.M410670200 15738001

[7] Bhattacharyya S Mandal D Sen GS Pal S Banerjee S Lahiry L. Tumor-Induced Oxidative Stress Perturbs Nfκb Activity Augmenting Tnfα-Mediated T Cell Death: Protection by Curcumin. Cancer Res (2007) 60:362–70. 10.1158/0008-5472 17210719

[8] Sa G Das T. Anti-Cancer Effects of Curcumin: Cycle of Life and Death. Cell Div (2008) 3:14. 10.1186/1747-1028-3-14 18834508 PMC2572158

[9] Klener P Otahal P Lateckova L Klener P. Immunotherapy Approaches in Cancer Treatment. Curr Pharm Biotechnol (2015) 16(9):771–81. 10.2174/1389201016666150619114554 26087990

[10] Pardoll D. Cancer and the Immune System: Basic Concepts and Targets for Intervention. Semin Oncol (2015) 42(4):523–38. 10.1053/j.seminoncol.2015.05.003 PMC5595144 26320058

[11] Hanson HL Donermeyer DL Ikeda H White JM Shankaran V Old LJ. Eradication of Established Tumors by CD8+ T Cell Adoptive Immunotherapy. Immunity (2000) 13(2):265–76. 10.1016/s1074-7613(00)00026-1 10981969

[12] Kalams SA Walker BD. The Critical Need for CD4 Help in Maintaining Effective Cytotoxic T Lymphocyte Responses. J Exp Med (1998) 188(12):2199–204. 10.1084/jem.188.12.2199 PMC2212425 9858506

[13] Knudson KM Hicks KC Luo X Chen JQ Schlom J Gameiro SR. M7824, a Novel Bifunctional Anti-PD-L1/TGFbeta Trap Fusion Protein, Promotes Anti-Tumor Efficacy as Monotherapy and in Combination With Vaccine. Oncoimmunology (2018) 7(5):e1584435. 10.1080/2162402X.2018.1426519 PMC5927523 29721396

[14] Albini A Bruno A Noonan DM Mortara L. Contribution to Tumor Angiogenesis From Innate Immune Cells Within the Tumor Microenvironment: Implications for Immunotherapy. Front Immunol (2018) 9:527. 10.3389/fimmu.2018.00527 29675018 PMC5895776

[15] Grivennikov SI Greten FR Karin M. Immunity, Inflammation, and Cancer. Cell (2010) 140(6):883–99. 10.1016/j.cell.2010.01.025 PMC2866629 20303878

[16] Panda AK Bose S Chakraborty S Kajal K Sa G. Intratumoral Immune Landscape: Immunogenicity to Tolerogenicity. Austin J Clin Immunol (2015) 2(1):1025.

[17] Attili I Karachaliou N Bonanno L Berenguer J Bracht J Codony-Servat J. STAT3 as a Potential Immunotherapy Biomarker in Oncogene-Addicted Non-Small Cell Lung Cancer. Ther. Adv Med Oncol (2018) 10:1758835918763744. 10.1177/1758835918763744 PMC5888808 29636826

[18] Rosenberg SA. A New Era for Cancer Immunotherapy Based on the Genes That Encode Cancer Antigens. Immunity (1999) 10:281–7. 10.1016/s1074-7613(00)80028-x 10204484

[19] Palmowski M Salio M Dunbar RP Cerundolo V. The Use of HLA Class I Tetramers to Design a Vaccination Strategy for Melanoma Patients. Immunol Rev (2002) 188:155–63. 10.1034/j.1600-065x.2002.18814.x 12445289

[20] Töpfer K Kempe S Müller N Schmitz M Bachmann M Cartellieri M. Tumor Evasion From T Cell Surveillance. J BioMed Biotechnol (2011) 2011:918471. 10.1155/2011/918471 22190859 PMC3228689

[21] Liu K Nussenzweig MC. Origin and Development of Dendritic Cells. Immunol Rev (2010) 234:45–54. 10.1111/j.0105-2896.2009.00879.x 20193011

[22] Hartmann E Wollenberg B Rothenfusser S Wagner M Wellisch D Mack B. Identification and Functional Analysis of Tumor-Infiltrating Plasmacytoid Dendritic Cells in Head and Neck Cancer. Cancer Res (2003) 63:6478–87. 14559840

[23] Ostuni R Kratochvill F Murray PJ Natoli G. Macrophages and Cancer: From Mechanisms to Therapeutic Implications. Trends Immunol (2015) 36:229–39. 10.1016/j.it.2015.02.004 25770924

[24] Dmitry IG Suzanne O Vincenzo B. Coordinated Regulation of Myeloidcells by Tumors. Nat Rev Immunol (2012) 12:253–69. 10.1038/nri3175

[25] Valkenburg KC de Groot AE Pienta KJ. Targeting the Tumor Stroma to Improve Cancer Therapy. Nat Rev Clin Oncol (2018) 15(6):366–81. 10.1038/s41571-018-0007-1 PMC5960434 29651130

[26] de Groot AE Roy S Brown JS Pienta KJ Amend SR. Revisiting Seed and Soil: Examining the Primary Tumor and Cancer Cell Foraging in Metastasis. MCR (2017) 15:361–70. 10.1158/1541-7786 PMC5380470 28209759

[27] Ruffell B Coussens LM. Macrophages and Therapeutic Resistance in Cancer. Cancer Cell (2015) 27:462–72. 10.1016/j.ccell.2015.02.015 PMC4400235 25858805

[28] Pienta KJ McGregor N Axelrod R Axelrod DE. Ecological Therapy for Cancer: Defining Tumors Using an Ecosystem Paradigm Suggests New Opportunities for Novel Cancer Treatments. Transl Oncol (2008) 1:158–64. 10.1593/tlo.08178 PMC2582164 19043526

[29] Munson JM Bellamkonda RV Swartz MA. Interstitial Flow in a 3D Microenvironment Increases Glioma Invasion by a CXCR4-Dependent Mechanism. Cancer Res (2013) 73:1536–46. 10.1158/0008-5472 23271726

[30] Xu K Cai YS Lu SM Li XL Liu L Li Z. Autophagy Induction Contributes to the Resistance to Methotrexate Treatment in Rheumatoid Arthritis Fibroblast-Like Synovial Cells Through High Mobility Group Box Chromosomal Protein 1. Arthritis Res Ther (2015) 17:374. 10.1186/s13075-015-0892-y 26702616 PMC4718027

[31] Baskar R Lee KA Yeo R Yeoh KW. Cancer and Radiation Therapy: Current Advances and Future Directions. Int J Med Sci (2012) 9:193–9. 10.7150/ijms.3635 PMC3298009 22408567

[32] Martinet L Garrido I Filleron T Le Guellec S Bellard E Fournie JJ. Human Solid Tumors Contain High Endothelial Venules: Association With T- and B-Lymphocyte Infiltration and Favorable Prognosis in Breast Cancer. Cancer Res (2011) 71:5678–87. 10.1158/0008-5472 21846823

[33] Bach JF. Regulatory T Cells Under Scrutiny. Nat Rev Immunol (2003) 3:189–98. 10.1038/nri1026 12658267

[34] Sprent J Surh CD. Knowing One’s Self: Central Tolerance Revisited. Nat Immunol (2003) 4:303–4. 10.1038/ni0403-303 12660725

[35] Curiel TJ. Tregs and Rethinking Cancer Immunotherapy. J Clin Invest (2007) 117(5):1167–74. 10.1172/JCI31202 PMC1857250 17476346

[36] Frydrychowicz M Boruczkowski M Kolecka-Bednarczyk A Dworacki G. The Dual Role of Treg in Cancer. Scand J Immunol (2017) 86(6):436–43. 10.1111/sji.12615 28941312

[37] Genard G Wera AC Huart C Le Calve B Penninckx S. Proton Irradiation Orchestrates Macrophage Reprogramming Through Nfκb Signaling. Cell Death Dis (2018) 9(7):728. 10.1038/s41419-018-0757-9 29950610 PMC6021396

[38] Shafabakhsh R Pourhanifeh MH Mirzaei HR Sahebkar A Asemi Z Mirzaei H. Targeting Regulatory T Cells by Curcumin: A Potential for Cancer Immunotherapy. Pharmacol Res (2019) 147:104353. 10.1016/j.phrs.2019.104353 31306775

[39] Sutmuller RP van Duivenvoorde LM van Elsas A Schumacher TN Wildenberg ME Allison JP. Synergism of Cytotoxic T Lymphocyte-Associated Antigen 4 Blockade and Depletion of CD25 (+) Regulatory T Cells in Antitumor Therapy Reveals Alternative Pathways for Suppression of Autoreactive Cytotoxic T Lymphocyte Responses. J Exp Med (2001) 194:823–32. 10.1084/jem.194.6.823 PMC2195955 11560997

[40] Liu VC Wong LY Jang T Shah AH Park I Yang X. Tumor Evasion of the Immune System by Converting CD4+CD25- T Cells Into CD4+CD25+ T Regulatory Cells: Role of Tumor Derived TGF-Beta. J Immunol (2007) 178:2883–92. 10.4049/jimmunol.178.5.2883 17312132

[41] Rosser EC Mauri C. Regulatory B Cells: Origin, Phenotype, and Function. Immunity (2015) 42:607–12. 10.1016/j.immuni.2015.04.005 25902480

[42] Zugazagoitia J Guedes C Ponce S Ferrer I Molina-Pinelo S Paz-Ares L. Current Challenges in Cancer Treatment. ClinicalTherapeutics (2016) 38(7):1551–66. 10.1016/j.clinthera.2016.03.026 27158009

[43] Weiner LM. Cancer Immunology for the Clinician. Clin Adv Hematol Oncol (2015) 13(5):299–306, 2015. 26352774

[44] Egen JG Kuhns MS Allison JP. CTLA-4: New Insights Into its Biological Function and Use in Tumor Immunotherapy. Nat Immunol (2002) 3:611–8. 10.1038/ni0702-611 12087419

[45] Parry RV Chemnitz JM Frauwirth KA lanfranco AR Braunstein I Kobayashi. CTLA-4 and PD-1 Receptors Inhibit T-Cell Activation by Distinct Mechanisms. Mol Cell Biol (2005) 25:9543–53. 10.1128/MCB.25.21.9543-9553.2005 PMC1265804 16227604

[46] Rozali EN Hato SV Robinson BW Lake RA Lesturhuis WJ. Programmed Death Ligand 2 in Cancer-Induced Immune Suppression. Clin Dev Immunol (2012) 2012:656340. 10.1155/2012/656340 22611421 PMC3350956

[47] Youngnak P Kozono Y Kozono H Iwai H Otsuki N Jin H. Differential Binding Properties of B7-H1 and B7-DC to Death-1. Biochem Biophys Res Commun (2003) 307:672–7. 10.1016/s0006-291x(03)01257-9 12893276

[48] Butte MJ Keir ME Phamduy TB Sharpe AH Freeman GJ. Programmed Death-1 Ligand 1 Interacts Specifically With the B7–1 Costimulatory Molecule to Inhibit T Cell Responses. Immunity (2007) 27:111–22. 10.1016/j.immuni.2007.05.016 PMC2707944 17629517

[49] Bose S Panda AK Mukherjee S Sa G. Curcumin and Tumor Immune-Editing: Resurrecting the Immune System. Cell Div (2015) 10:6. 10.1186/s13008-015-0012-z 26464579 PMC4603973

[50] Jagetia GC Aggarwal BB. “Spicing Up” of the Immune System by Curcumin. J Clin Immunol (2007) 27(1):19–35. 10.1007/s10875-006-9066-7 17211725

[51] Srivastava RM Singh S Dubey SK Misra K Khar A. Immunomodulatory and Therapeutic Activity of Curcumin. Int Immunopharmacol (2011) 11(3):331–41. 10.1016/j.intimp.2010.08.014 20828642

[52] Aggarwal BB Harikumar KB. Potential Therapeutic Effects of Curcumin, the Anti-Inflammatory Agent, Against Neurodegenerative, Cardiovascular, Pulmonary, Metabolic, Autoimmune and Neoplastic Diseases. Int J Biochem Cell Biol (2009) 41(1):40–59. 10.1016/j.biocel.2008.06.010 18662800 PMC2637808

[53] Bhattacharyya S Md Hossain SD Mohanty S Sen SG Chattopadhyay S Banerjee S. Curcumin Reverses T Cell-Mediated Adaptive Immune Dysfunctions in Tumor-Bearing Hosts. Cell Mol Immunol (2010) 7(4):306–15. 10.1038/cmi.2010.11 PMC4003225 20305684

[54] Churchill M Chadburn A Bilinski RT Bertagnolli MM. Inhibition of Intestinal Tumors by Curcumin is Associated With Changes in the Intestinalimmune Cell Profile. J Surg Res (2000) 89:169–75. 10.1006/jsre.2000.5826 10729246

[55] Zhao GJ Lu ZQ Tang LM Wu ZS Wang DW Zheng JY. Curcumin Inhibits Suppressive Capacity of Naturally Occurring CD4+CD25+regulatory T Cells in Mice *In Vitro* . Int Immunopharmacol (2012) 14(1):99–106. 10.1016/j.intimp.2012.06.016 22749847

[56] Ranjan D Chen C Johnston TD Jeon H Nagabhushan M. Curcumin Inhibits Mitogen Stimulated Lymphocyte Proliferation, NF-κb Activation, and IL-2 Signaling. J Surg Res (2004) 121(2):171–7. 10.1016/j.jss.2004.04.004 15501456

[57] Tomita M Kawakami H Uchihara JN Okudaira T Masuda M Takasu N. Curcumin Suppresses Constitutive Activation of AP-1 by Down-Regulation of JunD Protein in HTLV-1-Infected T-Cell Lines. Leuk Res (2006) 30(3):313–21. 10.1016/j.leukres.2005.08.004 16157375

[58] Hussain AR Al-Rasheed M Manogaran PS Al-Hussein KA Platanias LC Al Kuraya K. Curcumin Induces Apoptosis *via* Inhibition of PI3′-Kinase/AKT Pathway in Acute T Cell Leukemias. Apoptosis (2006) 11(2):245–54. 10.1007/s10495-006-3392-3 16502262

[59] Han SS Chung ST Robertson DA Ranjan D Bondada S. Curcumin Causes the Growth Arrest and Apoptosis of B Cell Lymphoma by Downregulation of Egr-1, C-Myc, Bcl-XL, NF-Kappa B, and P53. Clin Immunol (1999) 93:152–61. 10.1006/clim.1999.4769 10527691

[60] Ranjan D Siquijor A Johnston TD Wu G Nagabhuskahn M. The Effect of Curcumin on Human B-Cell Immortalization by Epstein–Barr Virus. Am Surg (1998) 64(1):47–51. 9457037

[61] Joe B Lokesh BR. Role of Capsaicin, Curcumin and Dietary N-3 Fatty Acids in Lowering the Generation of Reactive Oxygen Species in Rat Peritoneal Macrophages. Biochim Biophys Acta (1994) 1224(2):255–63. 10.1016/0167-4889(94)90198-8 7981240

[62] Kim K Ryu K Ko Y Park C. Effects of Nuclear factor-kappaB Inhibitors and Its Implication on Natural Killer T-Cell Lymphoma Cells. Br J Haematol (2005) 131(1):59–66. 10.1111/j.1365-2141.2005.05720.x 16173963

[63] Kim GY Kim KH Lee SH Yoon MS Lee HJ Moon DO. Curcumin Inhibits Immunostimulatory Function of Dendritic Cells: MAPKs and Translocation of NF-κb as Potential Targets. J Immunol (2005) 174:8116–24. 10.4049/jimmunol.174.12.8116 15944320

[64] Kahkhaie KR Mirhosseini A Aliabadi A Mohammadi A Mousavi MJ Haftcheshmeh SM. Curcumin: A Modulator of Inflammatory Signaling Pathways in the Immune System. Inflammopharmacology (2019) 27(5):885–900. 10.1007/s10787-019-00607-3 31140036

[65] Cundell DR Wilkinson F. Curcumin: Powerful Immunomodulator From Turmeric. Curr Immunol Rev (2014) 10(2):122–32. 10.2174/1573395510666141029233003

[66] Singh S Aggarwal BB. Activation of Transcription Factor NF-Kappa B Is Suppressed by Curcumin (Diferuloylmethane). J Biol Chem (1995) 270:24995–5000. 10.1074/jbc.270.42.24995 7559628

[67] Leonard WJ O’Shea JJ. Jaks and STATs: Biological Implications. Annu Rev Immunol (1998) 16:293–322. 10.1146/annurev.immunol.16.1.293 9597132

[68] O’shea JJ Holland SM Staudt LM. JAKs and STATs in Immunity, Immunodeficiency, and Cancer. N Engl J Med (2013) 368:161–70. 10.1056/NEJMra1202117 PMC7604876 23301733

[69] Guimarães MR Leite FRM Spolidorio LC Kirkwood KL Rossa C Jr. Curcumin Abrogates LPS-Induced Pro-Inflammatory Cytokines in RAW 264.7 Macrophages. Evidence for Novel Mechanisms Involving SOCS-1,-3 and P38 MAPK. Arch Oral Biol (2013) 58:1309–17. 10.1016/j.archoralbio.2013.07.005 PMC4030384 24011306

[70] Zhang L Wu C Zhao S Yuan D Lian G Wang X. Demethoxycurcumin, a Natural Derivative of Curcumin Attenuates LPS-Induced Pro-Inflammatory Responses Through Down-Regulation of Intracellular ROS-Related MAPK/NF-κb Signaling Pathways in N9 Microglia Induced by Lipopolysaccharide. Int Immunopharmacol (2010b) 10:331–8. 10.1016/j.intimp.2009.12.004 20018257

[71] Zhan Y Chen Y Liu R Zhang H Zhang Y. Potentiation of Paclitaxelactivity by Curcumin in Human Breast Cancer Cell by Modulating Apoptosis and Inhibiting EGFR Signaling. Arch Pharm Res (2014) 37(8):1086–95. 10.1007/s12272-013-0311-3 24318305

[72] Lee JH Chung IK. Curcumin Inhibits Nuclear Localization of Telomerase by Dissociating the Hsp90 Co-Chaperone P23 From hTERT. Cancer Lett (2010) 290(1):76–86. 10.1016/j.canlet.2009.08.026 19751963

[73] Shehzad A Lee YS. Molecular Mechanisms of Curcumin Action: Signal Transduction. Biofactors (2013) 39(1):27–36. 10.1002/biof.1065 23303697

[74] Lee AY Fan CC Chen YA Cheng CW Sung YJ Hsu CP. Curcumin Inhibits Invasiveness and Epithelial-Mesenchymal Transition in Oral Squamous Cell Carcinoma Through Reducing Matrix Metalloproteinase 2, 9 and Modulating P53-E-Cadherin Pathway. Integr Cancer Ther (2015) 14(5):484–90. 10.1177/1534735415588930 26036622

[75] Kunnumakkara AB Anand P Aggarwal BB. Curcumin Inhibits Proliferation, Invasion, Angiogenesis and Metastasis of Different Cancers Through Interaction With Multiple Cell Signaling Proteins. Cancer Lett (2008) 269(2):199–225. 10.1016/j.canlet.2008.03.009 18479807

[76] Tsvetkov P Asher G Reiss V Shaul Y Sachs L Lotem J. Inhibition of NAD(P)H:quinone Oxidoreductase 1 Activity and Induction of P53 Degradation by the Natural Phenolic Compound Curcumin. Proc Natl Acad Sci USA (2005) 102(15):5535–40. 10.1073/pnas.0501828102 PMC556252 15809436

[77] Pal S Choudhuri T Chattopadhyay S Bhattacharya A Datta GK Das T. Mechanisms of Curcumin-Induced Apoptosis of Ehrlich’s Ascites Carcinoma Cells. Biochem Biophys Res Commun (2001) 288(3):658–65. 10.1006/bbrc.2001.5823 11676493

[78] Karunagaran D Rashmi R Kumar TR. Induction of Apoptosis by Curcumin and its Implications for Cancer Therapy. Curr Cancer Drug Targets (2005) 5(2):117–29. 10.2174/1568009053202081 15810876

[79] Sanmamed MF Chen L. A Paradigm Shift in Cancer Immunotherapy: From Enhancement to Normalization. Cell (2018) 175(2):313–26. 10.1016/j.cell.2018.09.035 PMC6538253 30290139

[80] Schuster M Nechansky A Kircheis R. Cancer Immunotherapy. Biotechnol J (2006) 1(2):138–47. 10.1002/biot.200500044 16892244

[81] Ventola CL. Cancer Immunotherapy, Part 3: Challenges and Future Trends. P&T (2017) 42(8):514–21. PMC5521300 28781505

[82] Ellyard JI Simson L Parish CR. Th2-Mediated Anti-Tumor Immunity: Friend or Foe? Tissue Antigens (2007) 70:1–11. 10.1111/j.1399-0039.2007.00869.x 17559575

[83] Pal S Bhattacharyya S Choudhuri T Datta GK Das T Sa G. Amelioration of Immune Cell Number Depletion and Potentiation of Depressed Detoxification System of Tumor-Bearing Mice by Curcumin. Cancer Detect Prev (2005) 29(5):470–8. 10.1016/j.cdp.2005.05.003 16188398

[84] Zhao HM Xu R Huang XY Cheng SM Huang MF Yue HY. Curcumin Suppressed Activation of Dendritic Cells *via* JAK/STAT/SOCS Signal in Mice With Experimental Colitis. Front Pharmacol (2016) 25:455(7). 10.3389/fphar.2016.00455 PMC5122716 27932984

[85] deLeeuw RJ Kost SE Kakal JA Nelson BH. The Prognostic Value of FoxP3+ Tumor Infiltrating Lymphocytes in Cancer: A Critical Review of the Literature. Clin Cancer Res (2012) 18(11):3022–9. 10.1158/1078-0432.CCR-11-3216 22510350

[86] Pandiyan P Zheng L Ishihara S Reed J Lenardo MJ. CD41CD251Foxp31 Regulatory T Cells Induce Cytokine Deprivation-Mediated Apoptosis of Effector CD41 T Cells. Nat Immunol (2007) 8:1353–62. 10.1038/ni1536 17982458

[87] Tiemessen MM Mitchell TJ Hendry L Whittaker SJ Taams LS John S. Lack of Suppressive CD4+CD25+FOXP3+ T Cells in Advanced Stages of Primary Cutaneous T-Cell Lymphoma. J Invest Dermatol (2006) 126:2217–23. 10.1038/sj.jid.5700371 PMC2621310 16741512

[88] Barnett B Kryczek I Cheng P Zou W Curiel TJ. Regulatory T Cells in Ovarian Cancer: Biology and Therapeutic Potential. Am J Reprod Immunol (2005) 54:369–77. 10.1111/j.1600-0897.2005.00330.x 16305662

[89] Zhou G Drake CG Levitsky HI. Amplification of Tumor-Specific Regulatory T Cells Following Therapeutic Cancer Vaccines. Blood (2006) 107:628–36. 10.1182/blood-2005-07-2737 PMC1895618 16179369

[90] Gavin MA Rasmussen JP Fontenot JD Vasta V Manganiello VC Beavo JA. Foxp3-Dependent Programme of Regulatory T-Cell Differentiation. Nature (2007) 445:771–5. 10.1038/nature05543 17220874

[91] Liu Y Zou R Wang Z Wen C Zhang F Lin F. Exosomal KLF3-AS1 From hMSCs Promoted Cartilage Repair and Chondrocyte Proliferation in Osteoarthritis. Biochem J (2018) 475(22):3629–38. 10.1042/BCJ20180675 30341166

[92] Liao F Liu L Luo E Hu J. Curcumin Enhances Anti-Tumor Immune Response in Tongue Squamous Cell Carcinoma. Arch Oral Biol (2018) 92:32–7. 10.1016/j.archoralbio.2018.04.015 29751146

[93] Henricks LM Schellens JH Huitema AD Beijnen JH. The Use of Combinations of Monoclonal Antibodies in Clinical Oncology. Cancer Treat Rev (2015) 41(10):859–67. 10.1016/j.ctrv.2015.10.008 26547132

[94] Gul N van Egmond M. Antibody-Dependent Phagocytosis of Tumor Cells by Macrophages: A Potent Effector Mechanism of Monoclonal Antibody Therapy of Cancer. Cancer Res (2015) 75(23):5008–13. 10.1158/0008-5472.CAN-15-1330 26573795

[95] Teillaud JL. From Whole Monoclonal Antibodies to Single Domain Antibodies: Think Small. Methods Mol Biol (2012) 911:3–13. 10.1007/978-1-61779-968-6_1 22886242

[96] Chen R Chen B. Brentuximab Vedotin for Relapsed or Refractory Hodgkin’s Lymphoma. Drug Des Dev Ther (2015) 9:1729–33. 10.2147/DDDT.S82007 PMC4376183 25848209

[97] Chen R Hou J Newman E Kim Y Donohue C Liu X. CD30 Down-Regulation, MMAE Resistance, and MDR1 Up-Regulation Are All Associated With Resistance to Brentuximab Vedotin. Mol Cancer Ther (2015) 14(6):1376–84. 10.1158/1535-7163.MCT-15-0036 PMC4458438 25840583

[98] Barbet J Bardies M Bourgeois M Chatal JF Cherel M Davodeau F. Radiolabeled Antibodies for Cancer Imaging and Therapy. Methods Mol Biol (2012) 907:681–97. 10.1007/978-1-61779-974-7_38 22907380

[99] Buie LW Pecoraro JJ Horvat TZ Daley RJ. Blinatumomab: A First-in-Class Bispecific T-Cell Engager for Precursor B-Cell Acute Lymphoblastic Leukemia. Ann Pharmacother (2015) 49(9):1057–67. 10.1177/1060028015588555 26041811

[100] Alatrash G Jakher H Stafford PD Mittendorf EA. Cancer Immunotherapies, Their Safety and Toxicity. Expert Opin Drug Safety (2013) 12(5):631–45. 10.1517/14740338.2013.795944 23668362

[101] Thill M. New Frontiers in Oncology: Biosimilar Monoclonal Antibodies for the Treatment of Breast Cancer. Expert Rev Anticancer Ther (2015) 15(3):331–8. 10.1586/14737140.2015.993318 25539719

[102] Langone P Debata PR Dolai S Curcio GM Inigo Jdel R Raja K. Coupling to a Cancer Cell-Specific Antibody Potentiates Tumoricidal Properties of Curcumin. Int J Cancer (2012) 131(4):E569–78. 10.1002/ijc.26479 21989768

[103] Ye Z Qian Q Jin H Qian Q. Cancer Vaccine: Learning Lessons From Immune Checkpoint Inhibitors. J Cancer (2018) 9(2):263–8. 10.7150/jca.20059 PMC5771333 29344272

[104] Karlitepe A Ozalp O Avci CB. New Approaches for Cancer Immunotherapy. Tumor Biol (2015) 36(6):4075–8. 10.1007/s13277-015-3491-2 25934338

[105] Speiser DE Flatz L. Cancer Immunotherapy Drives Implementation Science in Oncology. Hum Vaccine Immunother (2014) 10(11):3107–10. 10.4161/21645515.2014.983000 PMC4514085 25625923

[106] Hurley LP Bridges CB Harpaz R Allison MA O' Leary ST Crane LA. Physician Attitudes Toward Adult Vaccines and Other Preventive Practices, United States, 2012. Public Health Rep (2016) 131(2):320–30. 10.1177/003335491613100216 PMC4765981 26957667

[107] Knutson KL Mittendorf EA. Cancer Vaccines in the New Era of Cancer Immunotherapy. Vaccine (2015) 33(51):7376. 10.1016/j.vaccine.2015.09.086 26432907

[108] Lu Y Miao L Wang Y Xu Z Zhao Y Shen Y. Curcumin Micelles Remodel Tumor Microenvironment and Enhance Vaccine Activity in an Advanced Melanoma Model. Mol Ther (2016) 24(2):364–74. 10.1038/mt.2015.165 PMC4817807 26334519

[109] Restifo NP Dudley ME Rosenberg SA. Adoptive Immunotherapy for Cancer: Harnessing the T Cell Response. Nat Rev Immunol (2012) 12(4):269–81. 10.1038/nri3191 PMC6292222 22437939

[110] Hawkins RE Gilham DE Debets R Eshhar Z Taylor N Abken H. Development of Adoptive Cell Therapy for Cancer: A Clinical Perspective. Hum Gene Ther (2010) 21(6):665–72. 10.1089/hum.2010.086 20408760

[111] Liu L Sommermeyer D Cabanov A Kosasih P Hill T Riddell SR. Inclusion of Strep-Tag II in Design of Antigen Receptors for T-Cell Immunotherapy. Nature Biotechnol (2016) 34(4):430–4. 10.1038/nbt.3461 PMC4940167 26900664

[112] Gill S Kalos M. T Cell-Based Gene Therapy of Cancer. Transl Res (2013) 161(4):365–79. 10.1016/j.trsl.2012.11.002 23246626

[113] Park TS Rosenberg SA Morgan RA. Treating Cancer With Genetically Engineered T Cells. Trends Biotechnol (2011) 29(11):550–7. 10.1016/j.tibtech.2011.04.009 PMC3193849 21663987

[114] Jackson HJ Brentjens RJ. Overcoming Antigen Escape With CAR T-Cell Therapy. Cancer Discovery (2015) 5(12):1238–40. 10.1158/2159-8290.CD-15-1275 PMC5536095 26637657

[115] Brudno JN Kochenderfer JN. Toxicities of Chimeric Antigen Receptor T Cells: Recognition and Management. Blood (2016) 127:3321–30. 10.1182/blood-2016-04-703751 PMC4929924 27207799

[116] Namuduri M Brentjens RJ. Medical Management of Side Effects Related to CAR T Cell Therapy in Hematologic Malignancies. Expert Rev Hematol (2016) 9:511–3. 10.1080/17474086.2016.1183479 PMC5539903 27139507

[117] Ahmed N Brawley VS Hegde M Robertson C Ghazi A Gerken C. Human Epidermal Growth Factor Receptor 2 (HER2) -Specific Chimeric Antigen Receptor-Modified T Cells for the Immunotherapy of HER2-Positive Sarcoma. J Clin Oncol (2015) 33:1688–96. 10.1200/JCO.2014.58.0225 PMC4429176 25800760

[118] Morgan RA Yang JC Kitano M Dudley ME Laurencot CM Rosenberg SA. Case Report of a Serious Adverse Event Following the Administration of T Cells Transduced With a Chimeric Antigen Receptor Recognizing ERBB2. Mol Ther (2010) 18:843–51. 10.1038/mt.2010.24 PMC2862534 20179677

[119] Chang YF Chuang HY Hsu CH Liu RS Gambhir SS Hwang JJ. Immunomodulation of Curcumin on Adoptive Therapy With T Cell Functional Imaging in Mice. Cancer Prev Res (2012) 5(3):444–52. 10.1158/1940-6207.CAPR-11-0308 22135043

[120] Aspeslagh S Marabelle A Soria JC Armand JP. Upcoming Innovations in Lung Cancer Immunotherapy: Focus on Immune Checkpoint Inhibitors. Chin Clin Oncol (2015) 4(4):48. 10.3978/j.issn.2304-3865.2015.12.06 26730760

[121] Makkouk A Weiner GJ. Cancer Immunotherapy and Breaking Immune Tolerance: New Approaches to an Old Challenge. Cancer Res (2015) 75(1):5–10. 10.1158/0008-5472.CAN-14-2538 25524899 PMC4286422

[122] Ciccarese C Alfieri S Santoni M Santini D Brunelli M Bergamini C. New Toxicity Profile for Novel Immunotherapy Agents: Focus on Immune-Checkpoint Inhibitors. Expert Opin Drug Metab Toxicol (2016) 12(1):57–75. 10.1517/17425255.2016.1120287 26565919

[123] Shore ND. Advances in the Understanding of Cancer Immunotherapy. BJU Int (2015) 116(3):321–9. 10.1111/bju.12692 24612369

[124] Bilusic M Madan RA Gulley JL. Immunotherapy of Prostate Cancer: Facts and Hopes. Clin Cancer Res (2017) 23:6764–70. 10.1158/1078-0432 PMC5690854 28663235

[125] Lynch TJ Bondarenko I Luft A Serwatowski P Barlesi F Chacko R. Ipilimumab in Combination With Paclitaxel and Carboplatin as First-Line Treatment in Stage IIIB/IV non-Small-Cell Lung Cancer: Results From a Randomized, Doubleblind, Multicenter Phase II Study. J Clin Oncol (2012) 30:2046–54. 10.1200/JCO.2011.38.4032 22547592

[126] Hossain DM Panda AK Manna A Mohanty S Bhattacharjee P. FoxP3 Acts as a Co-Transcription Factor With STAT3 in Tumor-Induced Regulatory T Cells. Immunity (2013) 39(6):1057–69. 10.1016/j.immuni.2013.11.005 24315995

[127] Fong L Engleman EG. Dendritic Cells in Cancer Immunotherapy. Annu Rev Immunol (2000) 18:245–73. 10.1016/j.it.2016.09.006 10837059

[128] Hayakawa T Yaguchi T Kawakami Y. Enhanced Anti-Tumor Effects of the PD-1 Blockade Combined With a Highly Absorptive Form of Curcumin Targeting STAT3. Cancer Sci (2020) 111(12):4326–35. 10.1111/cas.14675 PMC7734012 33006786

[129] Browning L Patel MR Horvath EB Tawara K Jorcyk CL. IL-6 and Ovarian Cancer: Inflammatory Cytokines in Promotion of Metastasis. Cancer Manag Res (2018) 10:6685–93. 10.2147/CMAR.S179189 PMC6287645 30584363

[130] Batra H Pawar S Bahl D. Curcumin in Combination With Anti-Cancer Drugs: A Nanomedicine Review. Pharmacol Res (2019) 139:91–105. 10.1016/j.phrs.2018.11.005 30408575

[131] Zangui M Atkin SL Majeed M Sahebkar A. Current Evidence and Future Perspectives for Curcumin and its Analogues as Promising Adjuncts to Oxaliplatin: State-of-the-Art. Pharmacol Res (2019) 141:343–56. 10.1016/j.phrs.2019.01.020 30641277

[132] Yue GG Kwok HF Lee JK Jiang L Wong EC Gao S. Combined Therapy Using Bevacizumab and Turmeric Ethanolic Extract (With Absorbable Curcumin) Exhibited Beneficial Efficacy in Colon Cancer Mice. Pharmacol Res (2016) 111:43–57. 10.1016/j.phrs.2016.05.025 27241019

[133] Chou TC. Theoretical Basis, Experimental Design, and Computerized Simulation of Synergism and Antagonism in Drug Combination Studies. Pharmacol Rev (2006) 58(3):621–81. 10.1124/pr.58.3.10 16968952

[134] Jia J Zhu F Ma X Cao Z Cao ZW Li Y. Mechanisms of Drug Combinations: Interaction and Network Perspectives. Nat Rev Drug Discovery (2009) 8(2):111–28. 10.1038/nrd2683 19180105

[135] Chen S Liang Q Xie S Liu E Yu Z Sun L. Curcumin Based Combination Therapy for Anti-Breast Cancer: From *In Vitro* Drug Screening to *In Vivo* Efficacy Evaluation. Front Chem Sci Eng (2016) 10(3):383–8. 10.1007/s11705-016-1574-2

[136] Basak SK Bera A Yoon AJ Morselli M Jeong C Tosevska A. A Randomized, Phase 1, Placebo-Controlled Trial of APG-157 in Oral Cancer Demonstrates Systemic Absorption and an Inhibitory Effect on Cytokines and Tumor-Associated Microbes. Cancer (2020) 126(8):1668–82. 10.1002/cncr.32644 32022261

[137] Tønnesen HH Másson M Loftsson T. Studies of Curcumin and Curcuminoids. XXVII. Cyclodextrin Complexation: Solubility, Chemical and Photochemical Stability. Int J Pharm (2002) 244(1-2):127–35. 10.1016/s0378-5173(02)00323-x 12204572

[138] Ireson CR Jones DJ Orr S Coughtrie MW Boocock DJ Williams ML. Metabolism of the Cancer Chemopreventive Agent Curcumin in Human and Rat Intestine. Cancer Epidemiol Biomarkers Prev (2002) 11(1):105–11. 11815407

[139] Ireson C Orr S Jones DJ Verschoyle R Lim CK Luo JL. Characterization of Metabolites of the Chemopreventive Agent Curcumin in Human and Rat Hepatocytes and in the Rat *In Vivo*, and Evaluation of Their Ability to Inhibit Phorbol Esterinduced Prostaglandin E2 Production. Cancer Res (2001) 61(3):1058–64. 11221833

[140] Sharma RA McLelland HR Hill KA Ireson CR Euden SA Manson MM. Pharmacodynamic and Pharmacokinetic Study of Oral Curcuma Extract in Patients With Colorectal Cancer. Clin Cancer Res (2001) 7(7):1894–900. 11448902

[141] Cheng AL Hsu CH Lin JK Hsu MM Ho YF Shen TS. Phase I Clinical Trial of Curcumin, a Chemopreventive Agent, in Patients With High-Risk or Pre-Malignant Lesions. Anticancer Res (2001) 21(4b):2895–900. 11712783

[142] Anand P Kunnumakkara AB Newman RA Aggarwal BB. Bioavailability of Curcumin: Problems and Promises. Mol Pharm (2007) 4(6):807–18. 10.1021/mp700113r 17999464

[143] Bisht S Feldmann G Soni S Ravi R Karikar C Maitra A. Polymeric Nanoparticle-Encapsulated Curcumin (“Nanocurcumin”): A Novel Strategy for Human Cancer Therapy. J Nanobiotechnol (2007) 5:3. 10.1186/1477-3155-5-3 PMC1868037 17439648

[144] Bonifacio BV Silva PB Ramos MA Negri KM Bauab TM Chorilli M. Nanotechnology-Based Drug Delivery Systems and Herbal Medicines: A Review. Int J Nanomed (2014) 9:1–15. 10.2147/IJN.S52634 PMC3862741 24363556

[145] Karthikeyan A Senthil N Min T. Nanocurcumin: A Promising Candidate for Therapeutic Applications. Front Pharmacol (2020) 11:487. 10.3389/fphar.2020.00487 32425772 PMC7206872

[146] Vashisht M Rani P Onteru SK Singh D. Curcumin Encapsulated in Milk Exosomes Resists Human Digestion and Possesses Enhanced Intestinal Permeability *In Vitro* . Appl Biochem Biotechnol (2017) 183(3):993–1007. 10.1007/s12010-017-2478-4 28466459

[147] Chidambaram M Krishnasamy K. Nanoparticulate Drug Delivery System to Overcome the Limitations of Conventional Curcumin in the Treatment of Various Cancers: A Review. Drug Delivery Lett (2014) 4(2):116–27. 10.2174/2210303103999131211110708

[148] Hossain DM Panda AK Chakrabarty S Bhattacharjee P Kajal K Mohanty S. MEK Inhibition Prevents Tumor-Shed Transforming Growth Factor-Beta-Induced T-Regulatory Cell Augmentation in Tumor Milieu. Immunology (2015) 144(4):561–73. 10.1111/imm.12397 PMC4368163 25284464

